# Ru Nanoparticles Ligated by an N‑Heterocyclic Carbene Derived from Uracil Nucleoside as Selective Antimicrobial Agents

**DOI:** 10.1021/acs.inorgchem.5c05649

**Published:** 2026-02-16

**Authors:** Adrián Sánchez, Luis A. M. Carrascosa, Giulia Romeo, Giulia Orsini, Yannick Coppel, Sarela Santamarina, Luis Rodríguez-Santiago, Xavier Solans-Monfort, Ana Petronilho, Luis M. Martínez-Prieto

**Affiliations:** † 518805 IIQ, Instituto de Investigaciones Químicas (CSIC-Universidad de Sevilla), Consejo Superior de Investigaciones Científicas, Avda. Americo Vespucio 49, 41092 Seville, Spain; ‡ 98819 Instituto de Tecnologia Química e Biológica António Xavier, Av. da Republica, 2780-157 Oeiras, Portugal; § CNRS, LCC (Laboratoire de Chimie de Coordination), 54919 Université de Toulouse, UPS, INPT, 205 route de Narbonne, BP 44099, F-31077 Toulouse Cedex 4, France; ∥ Department of Deep Microbiome Metabolomics, 28406 Leibniz Institute for Natural Product Research and Infection Biology (Leibniz-HKI), Beutenbergstrasse 11a, 07745 Jena, Germany; ⊥ Departament de Química, 16719 Universitat Autònoma de Barcelona, 08193 Cerdanyola del Vallès (Barcelona), Spain

## Abstract

Metal nanoparticles (MNPs) effectively combat pathogens due to their nonspecific toxicity, reducing the chance of bacterial resistance. Natural-based stabilizing ligands enhance the stability and targeting of MNPs while minimizing toxicity to human cells. In this context, ura-NHC-stabilized Ru NPs (**Ru@ura-NHC**) have been successfully synthesized following an organometallic approach, employing Ru­(COD)­(COT) as the metal precursor and, for the first time, a zwitterionic uracil-6-yl-imidazolium betaine (**ura-zwt**) as the NHC precursor. The mesomeric properties of **ura-zwt** enabled its use as an air-stable NHC precursor for the one-pot synthesis of **Ru@ura-NHC** with 100% atom economy and without byproduct formation. A combined theoretical/experimental study was conducted to investigate the coordination of the uracil-based NHC onto the Ru surface, indicating that the coordination of the neutral form (**ura-NHC**) is preferred over the zwitterionic form (**ura-zwt**). Evaluation of the antimicrobial properties of **Ru@ura-NHC** revealed that the coated Ru NPs exhibit selective activity against *Staphylococcus aureus*, which is beneficial as it minimizes off-target effects and reduces selective pressure for resistance.

## Graphical abstract

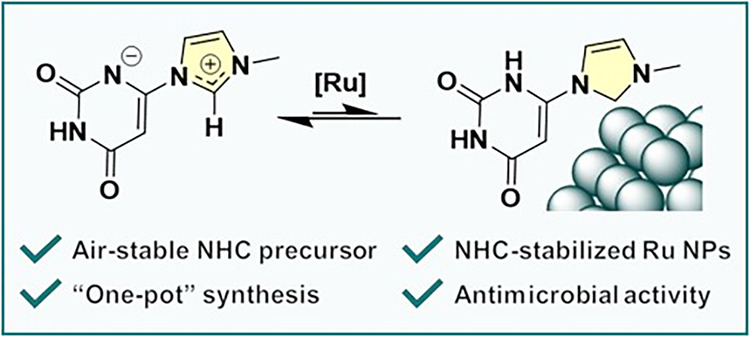

## Introduction

Metal nanoparticles (MNPs) exhibit unique chemical and physical properties that significantly differ from those of bulk materials.^1^ They have demonstrated remarkable utility in numerous fields, including biology, medicine, chemistry, materials science, and catalysis.^2–8^ MNPs are typically stabilized by polymers, which provide steric stabilization, or ligands, which offer electronic stabilization. These stabilization methods ultimately determine the size, stability and morphology of MNPs.^9^ Similar to organometallic complexes, stabilizing ligands can modify both the electronic and steric properties of MNPs, thereby influencing their surface chemistry.^10,11^ In this context, N-heterocyclic carbenes (NHCs) have emerged as effective ligands for MNP stabilization.^12,13^ Thanks to their unique electronic properties, NHCs offer several advantages as stabilizing ligands. For instance, their electron-donating ability allows them to form strong bonds with MNPs. Nevertheless, NHCs not only provide stabilization but also can modify the size, stability and reactivity of the MNPs.^14–16^ The nature and quantity of NHC used in the preparation of the MNPs have a significant impact on the physicochemical properties of NHC-stabilized MNPs. Depending on the N-substituent (electron donor/acceptor or bulky group) and the number of NHC equivalents employed as stabilizers, the resulting MNPs exhibit varying reactivity. As a result, NHCs have been utilized over the past few years to stabilize a wide range of MNPs made from different metals, including Ru,^17–19^ Pt,^20–22^ Au,^23^ Pd^14,24^ and Ir.^25^

While NHCs exhibit remarkable stability compared to classical carbenes or cyclic (alkyl)­(amino) carbenes,^26^ their high nucleophilicity makes them unstable in the presence of traces of water and open air.^27–29^ To address these challenges, bench-stable NHC precursors have become essential for reducing the moisture sensitivity of NHCs.^30^ Different NHC precursors have been reported, such as enetetramines,^31,32^ cyclic 2-thiones,^33^ 2-alkoxyimidazolidines,^34^ 2-(fluorophenyl)­imidazolines,^35^ imidazolium-2-carboxylates,^36^ and imidazolium-2-imidinates^37^ (Scheme 1a). The formation of the free NHCs from these precursors normally generates other byproducts and does not achieve complete atom economy. Inspired by Crabtreés synthesis of NHC-based organometallic complexes, Godard and co-workers used imidazolium carboxylates as bench-stable NHC precursors to synthesize NHC-stabilized Ni NPs through their decarboxylation in nonpolar solvents (Scheme 1b).^38^ In a comparable manner, this study reports the “one-pot” synthesis of NHC-stabilized Ru nanoparticles by generating an uracil-derived NHC (**ura-NHC**) from the corresponding air-stable zwitterionic betaine (**ura-zwt**). This novel approach avoids any basic pretreatment to generate the free NHC from the corresponding imidazolium salt, achieving this without forming byproducts and with 100% atom economy (Scheme 1c).

**Scheme 1:**
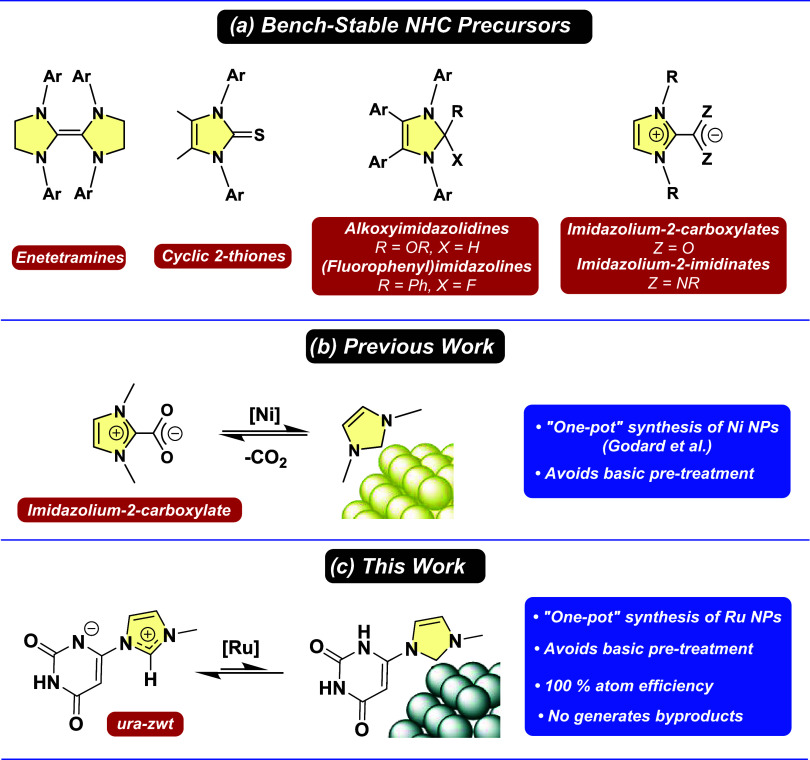
(a) Established Bench-Stable NHC Precursors; (b) One-Pot Synthesis of MNPs Using an Air-Stable Imidazolium Carboxylates and (c) Zwitterionic Betaine

Incorporating natural products such as uracil into a ligand system offers benefits such as accessible complex functional groups with unique structural and electronic properties, which can serve as effective recognition units, enhancing the selectivity and specificity of molecular interactions.^39^ The diversity of natural compounds provides a vast library of potential interactions and functionalities. Recent studies have shown that Ru NPs ligated by cholesterol-derived NHCs exhibit enhanced catalytic properties. The presence of the cholesterol moiety significantly influences the hydrogenation of aromatic compounds under mild conditions.^40^ The presence of natural compounds in stabilizing ligands can also enhance the biological activity of MNPs. MNPs are known to be effective against various pathogens due to their nonspecific bacterial toxicity mechanisms, which make it difficult for bacteria to develop resistance.^41,42^ They serve as a valuable alternative to existing antibiotics, facing significant resistance problems.^43–45^ Natural-based ligands can improve the stability, dispersibility, target specificity and reduce toxicity of MNPs to human cells while maintaining their antibacterial properties. In this regard, nucleobases are privileged scaffolds for developing MNP ligands, with the additional benefit of providing molecular recognition sites due to their ability to form base pairs.^39^ For example, uracil derivatives such as 5-fluorouracil are widely used as antifungals and have also been successfully employed as antibacterial agents.^46,47^ Specifically, uracil has been found to resensitize methicillin-resistant *Staphylococcus aureus* to antibiotics by modulating bacterial metabolism.^48^ Among the problematic pathogens, *Staphylococcus* species represent a significant challenge in terms of antibiotic resistance. Methicillin-resistant *S. aureus* has a global resistance rate of 27%, being classified by the World Health Organization (WHO) as a high-priority pathogen.^49^ The development of new drugs that target *S. aureus* is therefore crucial.

In this study, Ru NPs stabilized by an NHC derived from uracil (**Ru@ura-NHC**) were successfully synthesized following an organometallic approach and, for the first time, employing a zwitterionic uracil-6-yl-imidazolium betaine as NHC precursor (Scheme 1c). This ligand system provides the benefits of the uracil core in terms of molecular recognition and biocompatibility with the coordinating and stabilizing properties of the NHC. The presence and coordination mode of the stabilizing uracil-derived NHC were demonstrated by FT-IR, solid-state NMR, XPS and confirmed through DFT calculations. The antimicrobial efficiency of **Ru@ura-NHC** was evaluated against *S. aureus* and *Escherichia coli* and compared to reference materials, revealing a selective antimicrobial activity against the Gram-positive pathogen *S. aureus* due to a synergistic effect between the **ura-NHC** ligand and the Ru NPs.

## Experimental Section

### General Considerations and Starting Materials

Chemical procedures were performed under N_2_ atmosphere using Schlenk techniques and a glovebox. Schlenk flasks and Young ampules served as glassware. All reactions under H_2_ pressure were carried out using Fischer–Porter reactors.

Solvents were purified through distillation under N_2_ before utilization: *n*-pentane (Avantor VWR) and *n*-hexane (Avantor VWR) were distilled in the presence of metallic sodium; THF (Avantor VWR) was double-distilled (i) in the presence of CaCl_2_, and (ii) in the presence of metallic sodium; Methanol (Sigma-Aldrich) was distilled and purified using molecular sieve (3 Å); *n*-heptanol (Sigma-Aldrich) was dried with molecular sieve (3 Å). In all cases, O_2_ was removed from the solvent immediately before being used through N_2_ bubbling. Deuterated solvent: DMF-*d*_7_ (Eurisotop). Ru­(COD)­(COT) was purchased by Nanomeps Toulouse, and it was employed without further purification. Uracil-derived ligand 6-(3-methylimidazolio)-2,4­(3*H*)-pyrimidinedionate (**ura-zwt**) was synthesized using reported methods.^50–52^

### Methods

The methods used for characterization and analysis in this study are outlined as follows:

#### Transmission Electron Microscopy and High-Resolution Transmission Electron Microscopy (TEM/HRTEM)

Performed by the electron microscopy service of CITIUS at the University of Seville (US). A drop of isolated Ru NPs suspension in THF was deposited on a copper grid, and TEM/HRTEM images of the obtained Ru NPs were acquired with a FEI Talos S200 electron microscope operating at 200 kV with a point resolution of 2.5 Å. Particle size distribution was calculated by measuring the size of 200 particles using ImageJ and Origin software.

#### X-ray Photoelectron Spectroscopy (XPS)

Performed by the XPS service of the Seville Institute of Materials Science (ICMS, CSIC-US); Phoibos Hemispherical Energy Analyzer 100 1D-DLD. The detector is a 1D Dual Delay Line Detector 1D-DLD43–2–100. The X-ray source was an X-ray source XR50 with a nonmonochromated Al Kα line at 1486.71 eV and Mg Kα 1253.64 eV anode. The zones were recorded with Mg. Emission current of 15 mA and emission voltage of 11.5 kV. Chamber pressure during data collection: ∼10 mbar. Step size (eV): General 0.5 and elements 0.1. Passage energy (eV): General 50 and elements 30.

#### Fourier-Transformed Infrared Spectroscopy (FT-IR)

Measurements were performed on a Bruker Tensor 27 spectrometer in the range 4000–450 cm^–1^, with a resolution of 4 cm^–1^. Experiments were carried out in attenuated total reflectance mode (ATR).

#### Inductively Coupled Plasma-Optical Emission Spectrometry (ICP-OES)

Conducted by the ICP-OES service at CITIUS, University of Seville. Plasma Atomic Emission Spectrometer, SciAps Z-300, using a calibration curve between 0 and 100 ppm of Ru. The samples were digested using a mixture of HCl (3 mL), HNO_3_ (1 mL), and HBF_4_ (2 mL), and heated to 230 °C for 15 min in a microwave oven operating at 1800 W.

#### Nuclear Magnetic Resonance (NMR)

Recorded on a Bruker Avance III 400 spectrometer. The solvent resonances of ^1^H were used as internal standards, and chemical shifts are reported regarding TMS. Assignments were routinely performed by using ^1^H NMR.

#### Magic Angle Spinning NMR (MAS NMR)

NMR experiments were recorded at the LCC on a Bruker Avance 400 III HD spectrometer operating at magnetic fields of 9.4 T. Samples were packed into 3.2 mm zirconia rotors and were rotated at a MAS frequency of 13 kHz at 298 K. Ramped cross-polarization (CP) ^1^H → ^13^C were recorded with a 2 s recovery delay and 2 ms contact times combined with a Hahn-echo block synchronized with the spinning rate. Chemical shifts were externally referenced to liquid TMS.

#### Elemental Analysis

Performed by the Elemental Analysis Service of the Institute of Chemical Research (IIQ, CSIC-US). Measurements were performed on a LECO TRUSPEC CHNS at 1050 °C with He as the carrier gas. This instrument includes an afterburner equipped with a copper stick operating at 850 °C, which reduces elements to their elemental state. Carbon, hydrogen, and sulfur are detected using infrared detection methods, while nitrogen is measured using a thermal conductivity detector.

### Synthesis of Ruthenium Nanoparticles

#### Ru@ura-NHC

In a Schlenk flask, 31 mg of **ura-zwt** (0.16 mmol, 0.5 equiv) was dissolved in 10 mL of THF/MeOH (3:1 v/v) and magnetically stirred until the solid was fully dissolved. Parallelly, 100 mg of Ru­(COD)­(COT) (0.32 mmol, 1.0 equiv) were dissolved in 5 mL of THF in a Fischer–Porter reactor. Then, the **ura-zwt** solution was transferred to the Fischer–Porter containing the Ru­(COD)­(COT) solution via cannula. To prevent an undesired complex formation, the transfer was carried out by placing the Fischer–Porter in an acetone bath at −60 °C, while avoiding any agitation. The Fischer–Porter was then pressurized with 3 bar of H_2_ and left to react under stirring overnight at room temperature. After the formation of the Ru NPs (confirmed by the presence of a black suspension), the Fischer–Porter was depressurised. The solvent was then evaporated under vacuum. After that, the Ru NPs were washed three times with *n*-pentane. Finally, **Ru@ura-NHC** was obtained as a black powder after drying it under vacuum for 2 h (53 mg, yield 78%).

#### Ru@ura-NHC_(hept)_

First, *n*-heptanol-functionalized Ru NPs (**Ru@hept**) were prepared through a previously described synthesis route.^53^ Specifically, 100 mg of Ru­(COD)­(COT) (0.32 mmol, 1.0 equiv) were added to a Fischer–Porter and dissolved in 3–5 mL of *n*-heptanol. Then, the Fischer–Porter was pressurized with 3 bar of H_2_ and it was left to react under stirring for 1 h at room temperature. Once the **Ru@hept** were formed (confirmed by the presence of a back suspension), the Fischer–Porter was depressurised, and a solution of 31 mg of **ura-zwt** (0.16 mmol, 0.5 equiv) in THF/MeOH (3:1 v/v) was transferred via canula. Then, the mixture was left to react under magnetic stirring for 24 h. After that, the solvent was evaporated under vacuum and heating at 70 °C. Then the Ru NPs were washed three times with *n-*hexane. Finally, **Ru@ura-NHC**_**(hept)**_ was obtained as a black powder after drying it under vacuum for 2h (41 mg, 51% yield).

## Results and Discussion

### Synthesis and Characterization of Ru NPs

Zwitterionic uracil-6-yl-imidazolium betaine (**ura-zwt**) was prepared according to a previously reported procedure. It involves the reaction of the corresponding imidazolium salt with sodium acetate.^50,51^ This zwitterionic betaine belongs to the class of cross-conjugated heterocyclic mesomeric betaines and exists in equilibrium with the corresponding neutral tautomeric form (Scheme 2). It was then used as a carbene precursor to stabilize Ru NPs through an organometallic approach. MNPs were prepared via the controlled decomposition of Ru­(COD)­(COT) (COD: cyclooctadiene; COT: cyclooctatriene) in a mixture of THF:MeOH (3:1) at 3 bar of H_2_ in the presence of 0.5 equiv of **ura-zwt**, which acted as NHC precursor and controlled the coalescence of Ru atoms (Scheme 2a). The synthesis of NHC-stabilized MNPs through the organometallic synthesis normally requires either the prior isolation of the NHC,^19^ or the *in situ* formation of the free carbene by deprotonation with KO*t*Bu.^12,16,18,21^ The main advantage of using zwitterionic betaines as an NHC source is that they serve as bench-stable NHC precursors or “masked” NHC, mitigating storage and handling challenges associated with the sensitivity of carbenes to air. The mesomeric effect of the zwitterionic betaine allows the equilibrium to shift toward the NHC tautomer when trapping reagents, such as metal centers, are present.^50^ The presence of ruthenium atoms generated from the controlled decomposition of Ru­(COD)­(COT) during the synthesis shifts this equilibrium to the NHC that ultimately stabilizes the metal nanoparticle (Scheme 2a). This novel approach enables the use of **ura-zwt** as NHC precursor for MNP stabilization, avoiding the need to either isolate the free NHC or generate it in situ with a strong base. In conjunction with Godard́s work,^38^ it represents an elegant method for coordinating NHCs to MNP surfaces. The nanoparticles synthesized through this procedure will be named **Ru@ura-NHC**, hereafter.

**Scheme 2:**
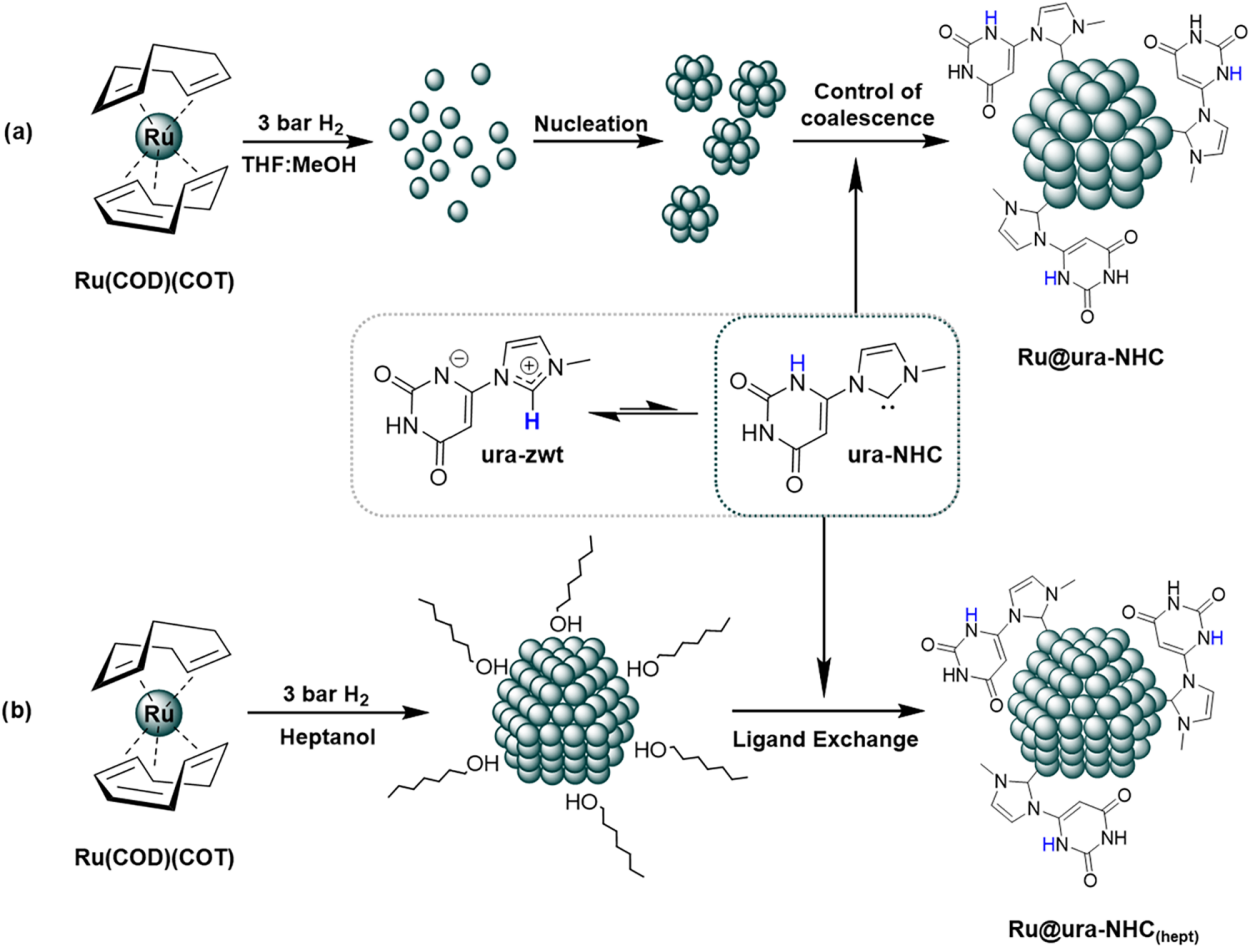
Synthesis of (a) **Ru@ura-NHC** and (b) **Ru@ura-NHC**_**(hept)**_ Using the Zwitterionic Betaine (**ura-zwt**) as a Bench-Stable NHC Precursor

The efficiency of **ura-zwt** in performing a ligand exchange process to produce larger Ru NPs (∼3 nm) stabilized with the same uracil-derived ligand was also explored. This allowed us to evaluate how the size of the Ru NPs affects their antibacterial activity (*vide infra*). Weakly stabilized Ru NPs of approximately 3 nm in size were prepared by decomposing Ru­(COD)­(COT) under 3 bar of H_2_ in *n-*heptanol, as previously described.^53^ In this case, *n-*heptanol acts as both a solvent and stabilizer, controlling the atom coalescence necessary for the formation of the MNP. Then, a THF/MeOH (3:1) solution of **ura-zwt** was added to a dispersion of these nanoparticles in *n-*heptanol (Scheme 2b).^54^ In this case, the equilibrium also shifts toward **ura-NHC** when *n-*heptanol-stabilized Ru nanoparticles are present. This leads to the formation of 3 nm nanoparticles covered with **ura-NHC** (**Ru@ura-NHC**_**(hept)**_), demonstrating the potential of **ura-zwt** as NHC precursor in ligand exchange processes.^55^

TEM analyses of both samples, **Ru@ura-NHC** and **Ru@ura-NHC**_**(hept)**_, revealed the formation of spherical and well-distributed Ru NPs with mean diameters of 2.1 ± 0.2 nm and 3.2 ± 0.5 nm, respectively (Figure 1a,c). As expected, **Ru@ura-NHC**_**(hept)**_ exhibits the same mean size as the parent *n-*heptanol-stabilized Ru NPs^53^ but displays ura-NHC ligands coordinated to its surface (*vide infra*). High-resolution TEM (HRTEM) images of both **Ru@ura-NHC** and **Ru@ura-NHC**_**(hept)**_ confirm the presence of crystalline hcp-Ru NPs (hexagonal close-packed), which is characteristic of metallic ruthenium (Figure 1b,1d).

**Figure 1:**
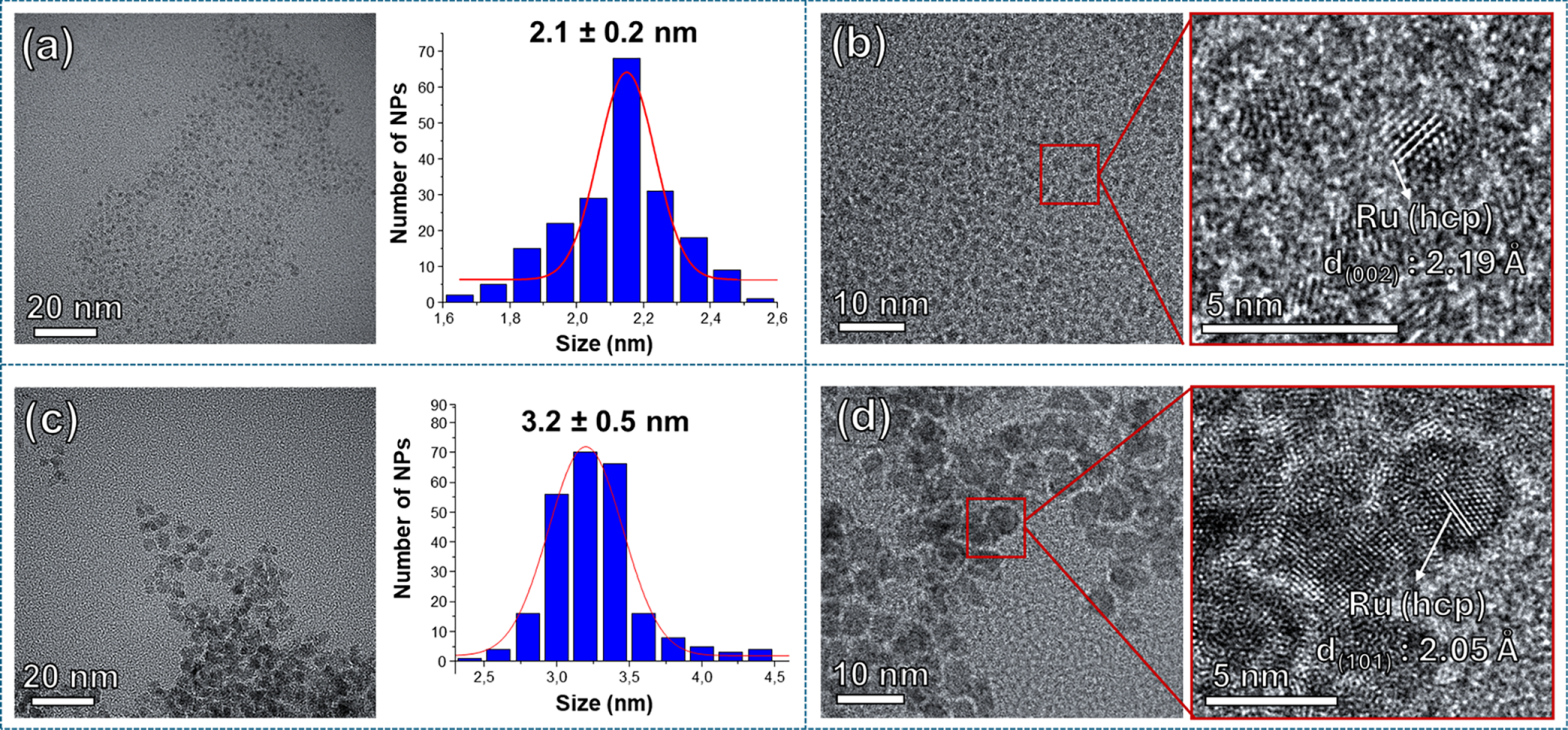
TEM micrographs and size histograms of (a) **Ru@ura-NHC** and (c) **Ru@ura-NHC**_**(hept)**_. HRTEM images and zoomed areas of (b) **Ru@ura-NHC** showing a lattice fringe spacing of 2.19 Å that corresponds to the Ru(002) crystal plane of metallic Ru and (d) **Ru@ura-NHC**_**(hept)**_ displaying a *d*-spacing of 2.05 Å that belongs to the Ru(111) plane. Both HRTEM images indicate the presence of crystalline Ru NPs retaining the hcp structure.

The amount of ruthenium was determined using Inductively Coupled Plasma Optical Emission Spectroscopy (ICP-OES), showing metal contents of 47.5 and 40.5 wt % for **Ru@ura-NHC** and **Ru@ura-NHC**_**(hept)**_, respectively. The estimated number of Ru surface atoms [Ru(s)] is not enough to coordinate all ligand molecules to the Ru surface, as the Ru(s)/ligand ratios are below one (see SI, Section S.2., Table S1). As previously observed for other MNPs stabilized with similar ligands,^56,57^ the excess of ligands may be located in a second coordination sphere, likely bonded by base pairing interactions. In fact, the ability of the uracil ligands to undergo self-base pairing is well-known.^39^ In the specific case of **ura-zwt**, its ability to self-base pair was measured by analyzing the ^1^H NMR spectra of **ura-zwt** solutions at different concentrations in DMSO-*d*_*6*_. Upon increasing the concentration of **ura-zwt** from 20 mM to 100 mM, a slight downfield shift of the NH signal was observed, which supports the formation of the **ura-zwt:ura-zwt** base pair (see SI, section S.3, Figures S1 and S2). The interaction of the **ura-zwt** with both **Ru@ura-NHC** and **Ru@ura-NHC**_**(hept)**_ was also examined by ^1^H NMR (see SI, Section S.3.2, Figures S3–S6). Yet, addition of 10 mg of **Ru@ura-NHC** to a DMSO or DMF solution of **ura-zwt** (20 mM) did not lead to any variation of the ^1^H NMR spectra. A similar outcome was obtained with **Ru@ura-NHC**_**(hept)**_.

### Coordination Studies

Spectroscopic techniques such as FT-IR, MAS NMR and XPS have proven effective in investigating the coordination modes of stabilizing ligands on MNP surfaces.^58–63^ The presence of the ligand on the Ru surface was first indicated by Fourier-transform infrared spectroscopy (FT-IR). By comparing the IR spectra of the zwitterionic betaine ligand with those of the ruthenium nanoparticles (see SI, Section S.4., Figure S7), the characteristic strong C–N bands around 1600 cm^–1^ in both spectra were observed.^56^ This indicates that the ligands are coordinated to the surface of the Ru NPs. New peaks were also detected at 1925–1945 cm^–1^ in both spectra. These signals can be attributed to CO coordinated to the Ru surface, resulting from the partial decarbonylation of THF used during the synthesis.^57^ These CO bands typically appear between 1900 and 1950 cm^–1^, as previously observed.^16,40,64^

Solid-state MAS NMR confirmed the existence of the ligand on the Ru surface. Most of their signals could be identified in the ^1^H → ^13^C CP-MAS NMR spectra recorded for purified **Ru@ura-NHC** and **Ru@ura-NHC**_**(hept)**_ samples (see SI, Section S.5., Figure S8). The intense signals corresponding to the methyl groups around 35 ppm are clearly observed on both spectra. Broad resonances between 110 and 140 ppm are related to the imidazolin-2-ylidene backbone, and the uracil carbon peaks are between 150 and 170 ppm in both systems (see SI, Section S.5., Figure S8b,c). The signals at 10 and 70 ppm observed in the **Ru@ura-NHC**_**(hept)**_ spectrum can be related to some remaining heptanol at the Ru surface after the ligand exchange process.^53^ The signals of the ligand are sharper for **Ru@ura-NHC**_**(hept)**_ compared to **Ru@ura-NHC**. This suggests that the ligands in **Ru@ura-NHC**_**(hept)**_ are either more ordered than those in **Ru@ura-NHC**, or that the presence of *n-*heptanol, along with the larger radius of nanoparticle curvature, allows for greater freedom of movement.

X-ray photoelectron spectroscopy (XPS) is a common technique used to analyze the metal composition and oxidation states of surface catalysts. It has also been used to study the coordination modes of surface ligands on ligand-stabilized MNPs.^56,64–66^ XPS was then employed to investigate how **ura-zwt** coordinates to the Ru nanoparticle surface. The N 1s region of **ura-zwt** displays an asymmetric broad peak ranging from 403 to 394 eV, which can be deconvoluted into three distinct contributions with relative intensities of 2:1:1 (Figure 2a). The main contribution at 399.8 eV is attributed to the strongly bound electrons of the nitrogen atoms in the imidazolium ring (Figure 2a, blue). The contribution at 397.9 eV can be assigned to the NH group of the uracil fragment (Figure 2a, orange). The third peak at 396.5 eV derives from the N atom of the uracil that bears a negative charge (Figure 2a, green). The N 1s regions of **Ru@ura-NHC** and **Ru@ura-NHC**_**(hept)**_ show symmetric peaks at 399.5 and 399.8 eV, respectively (Figure 2b and see SI, Section S.6., Figure S9), which are in agreement with reported values for NHC-stabilized MNPs.^40,67,68^ Compared to the asymmetric broad peak of the **ura-zwt** used as NHC precursor (from ∼395 to ∼403 eV), a notable change is visible. The symmetrical peaks of **Ru@ura-NHC** and **Ru@ura-NHC**_**(hept)**_ are attributed to the overlapped signals of the N atoms of the imidazolin-2-ylidene ring together with the signal corresponding to the NH groups of the uracil moiety. These findings confirm that the ligand is coordinated to the nanoparticle in its carbene form (i.e., **NHC-ura**), as illustrated in Scheme 2 and Figure 2, indicating that the mesomeric effect of **ura-zwt** allows its use as NHC precursor.^50^

**Figure 2:**
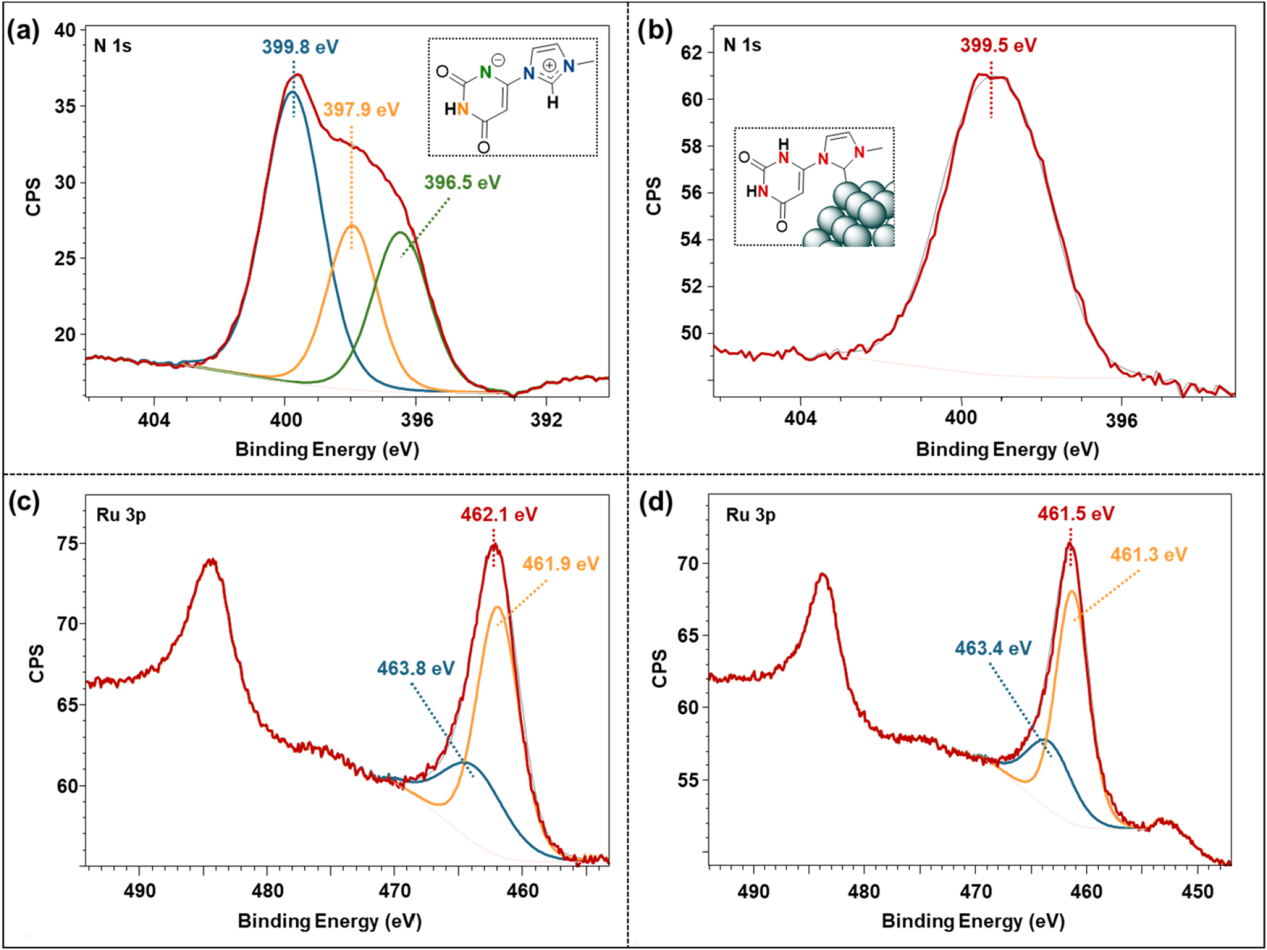
X-ray photoelectron spectroscopy (XPS) of the N 1s signals of (a) **ura-zwt** and (b) **Ru@ura-NHC**. XPS of the Ru 3p signals of (c) **Ru@ura-NHC** and (d) **Ru@ura-NHC**_**(hept)**_.

The oxidation states of **Ru@ura-NHC** and **Ru@ura-NHC**_**(hept)**_ were also investigated by XPS. Due to the overlap of the Ru 3d and C 1s signals, which complicated the interpretation and deconvolution, the corresponding Ru 3p regions were instead analyzed. Figure 2c,2d display a Ru 3p5/2 peak at 461.5–462.1 eV, which can be deconvoluted into two distinct components. The main component located at 461.9–461.3 eV, is attributed to Ru(0),^69^ indicating that **Ru@ura-NHC** and **Ru@ura-NHC**_**(hept)**_ are predominantly composed of metallic ruthenium. The secondary component at 463.8 to 463.4 eV corresponds to RuO_2_ (28–31%), likely formed during the sample preparation for XPS in air.

### DFT Calculations

The spectroscopy studies, along with the crucial role of theoretical calculations in clarifying experimental data, make combined experimental and theoretical studies ideal for determining the coordination mode of stabilizing ligands and for enhancing the understanding of chemical processes at the MNP surface.^62,64,70,71^ To provide further support on the adsorption mode of the uracil-bearing N-heterocyclic carbene on the Ru NPs, the adsorption of one and several ligands on the 1 nm large Ru_57_H_61_ nanoparticle model was analyzed. For the adsorption of one ligand, the seven different sites reported in Figure 3a for neutral, zwitterionic and abnormal isomers were considered (Figure 3c). Two anionic forms arising from transferring one acid H of the ligand to the surface were also considered in the same seven positions (Figure 3c). In these cases, the system is still globally neutral (Lig^–^-Ru_57_H_62_) as the number of atoms does not change. Calculations for the adsorption of two, three and four ligands were also performed for the most stable neutral, zwitterionic and anionic forms. For that, the additional ligands were located in the equivalent most favorable sites. The adsorption of eight and ten ligands was also explored for the neutral form only, following the same strategy. Since the computational model is smaller than the experimentally synthesized nanoparticle the adsorption of one ligand on a H-terminated crystalline Ru(100) surface (Figure 3b) was also analyzed. This system serves as a representative model of a large nanoparticle with predominant nondefective crystalline surfaces. The zwitterionic form is the most favorable structure of an isolated ligand in solution (Figure 3c), and this structure serves as reference for computing the ligand adsorption energies.

**Figure 3:**
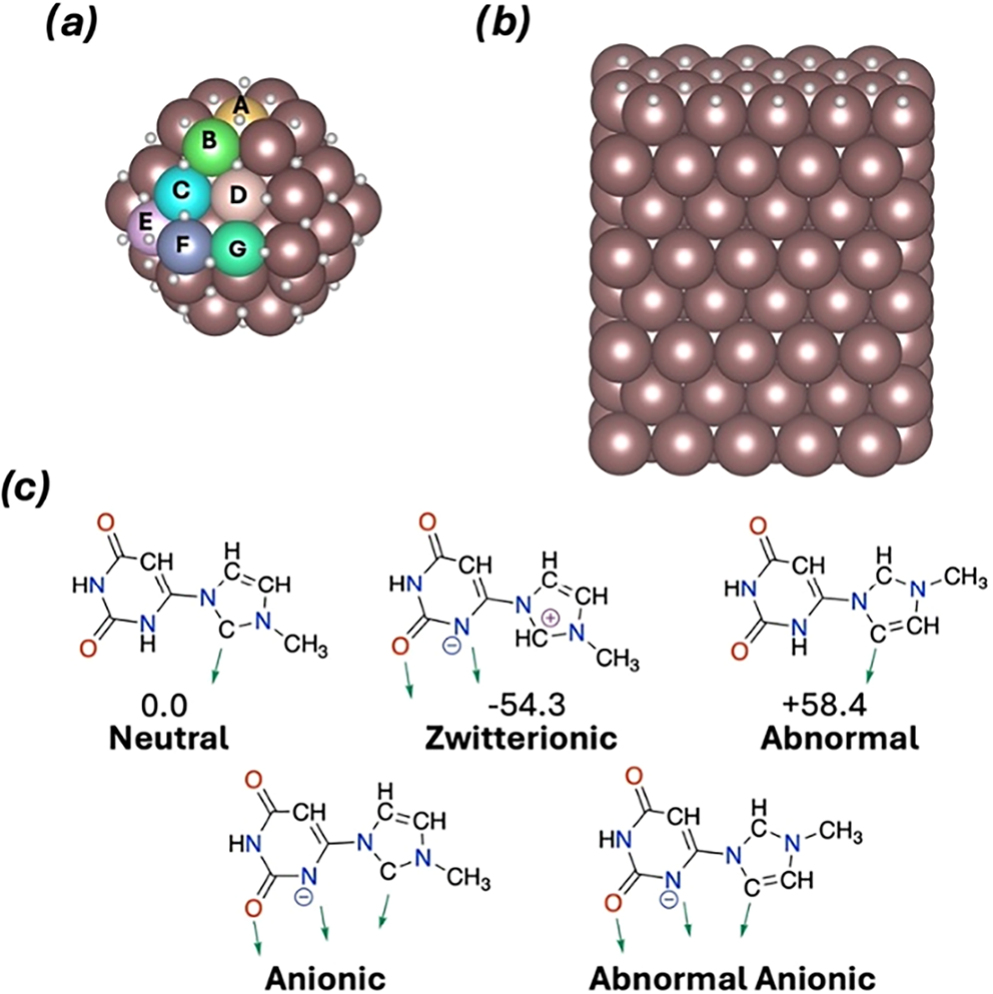
(a, b) Models and (c) coordinative modes used in DFT simulations. Relative energies with respect to the neutral form in kJ mol^–1^. Green arrows show the preferred coordination sites.

### 1 Ligand Adsorption

Figure 4 shows the most stable structure of the neutral, zwitterionic, abnormal, anionic and abnormal-anionic forms on Ru_57_H_62_, the adsorption energy and the ligand–metal distance. All other computed structures can be found in the Supporting Information together with those of the crystalline model (see SI, Section S.7., Figures S10–S18). Regarding the ligand in its neutral form and independently of the isomeric form, the adsorption is stronger on edge (C, E and G) and corner sites (B and F) and weaker on more coordinated atoms (sites A and D) (Figures S10–S14). The adsorption on the nanoparticle always occurs through a direct Ru-L coordination between either the C carbons of the NHC moiety or the N and O centers of uracil. This contrasts with the results for the crystalline model. In this case, the most stable adsorption mode shows the uracil-bearing N-heterocyclic carbene parallel to the surface, interacting mainly through van der Waals interactions (Figure S11). The adsorption energy on nanoparticle defective sites is almost 100 kJ mol^–1^ higher than the adsorption through van der Waals interactions of the flat surfaces (228 vs 136 kJ mol^–1^), suggesting that even in the 2–3 nm large nanoparticles, most of the coordinated ligands will be located in edges and corners. This is the consequence of two effects. The smaller size and convex morphology of the nanoparticle decrease the dispersive stabilizing interaction between the ligand and the material. The presence of poorly coordinated metals allows stronger interactions with the ligand. Since the coordinative mode becomes significantly preferred, the individual ability of each ligand form to interact with ruthenium strongly influences the most stable structure. The interaction through the carbon of the NHC ligands is stronger than that through N and O of uracil and the neutral form becomes preferred over the zwitterionic form. Special mention should be made regarding the stabilization of the anionic forms. The H transfer to Ru_57_H_61_ forming Ru_57_H_62_ is favorable^72^ and the resulting anionic structures (either normal or abnormal) can act as tridentate ligands occupying three coordination sites of an edge. This leads to significant adsorption energy that is only marginally lower than that of the neutral form. Overall, calculations suggest that the defective nanoparticle sites stabilize the neutral form with respect to the zwitterionic and anionic structures, which agrees with the interpretation of the XPS spectra. Zwitterionic form is preferred when interacting through van der Waals. However, the size of the ligand (around 0.8 nm), advocates that larger nanoparticles than those synthesized experimentally would be necessary to see this form as major coordination mode.

**Figure 4:**
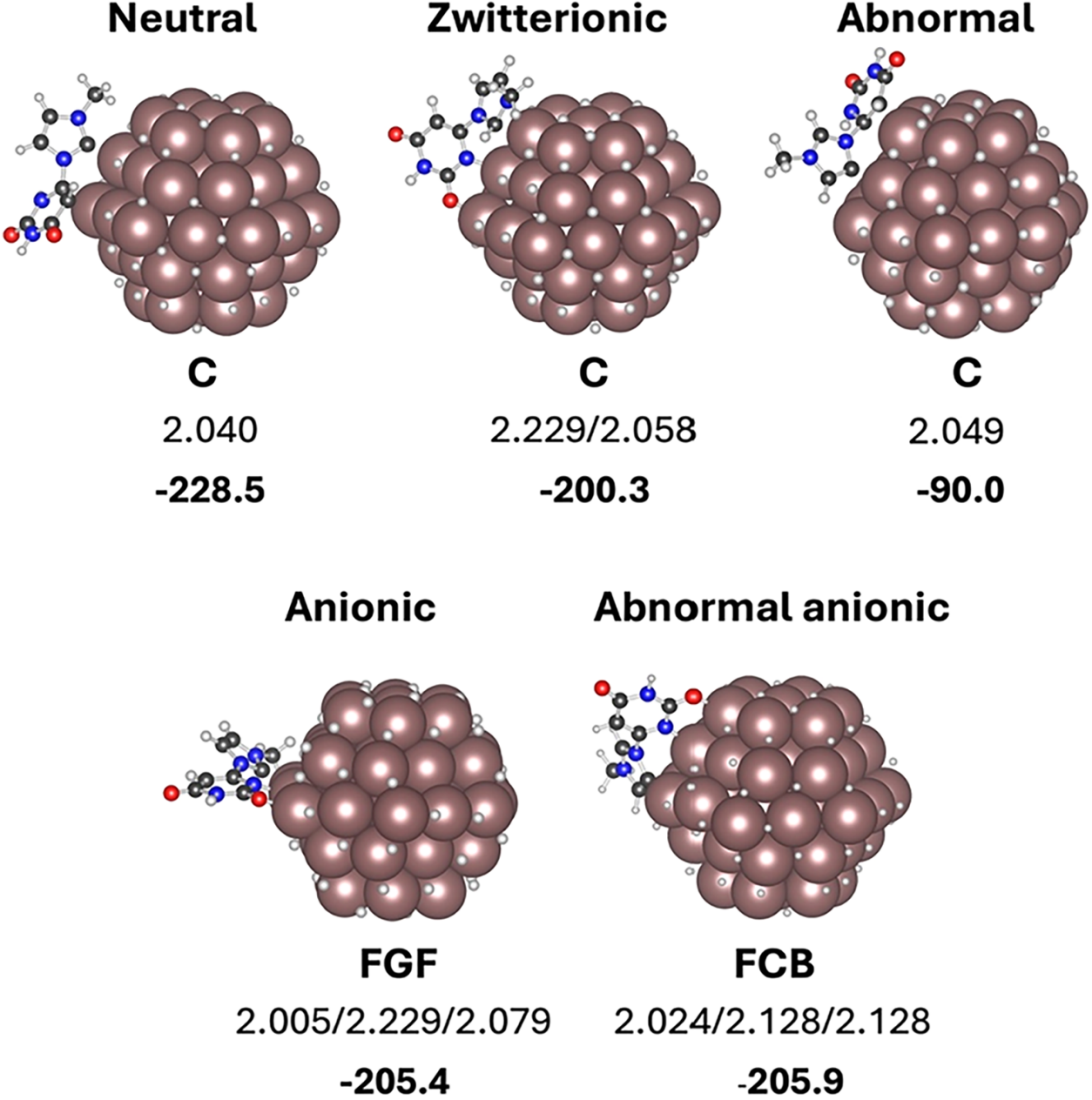
Most stable adsorption modes on the Ru nanoparticle. Ru-Ligand distances in Å and relative energies with respect to the Ru_57_H_61_ NP and the isolated zwitterionic form of the ligand in kJ mol^–1^.

### Several Ligand Adsorption

To study whether the preference for the neutral form is maintained or enhanced when increasing the number of ligands, additional calculations with 2, 3, and 4 ligands presenting either the neutral, the zwitterionic and the anionic forms were performed. For the most favorable neutral form, the adsorption of 8 and 10 ligands was also considered (Figure 5). Results for the adsorption of several neutral, zwitterionic and anionic ligands are summarized in Figures 5, S11 and S12, respectively.

**Figure 5:**
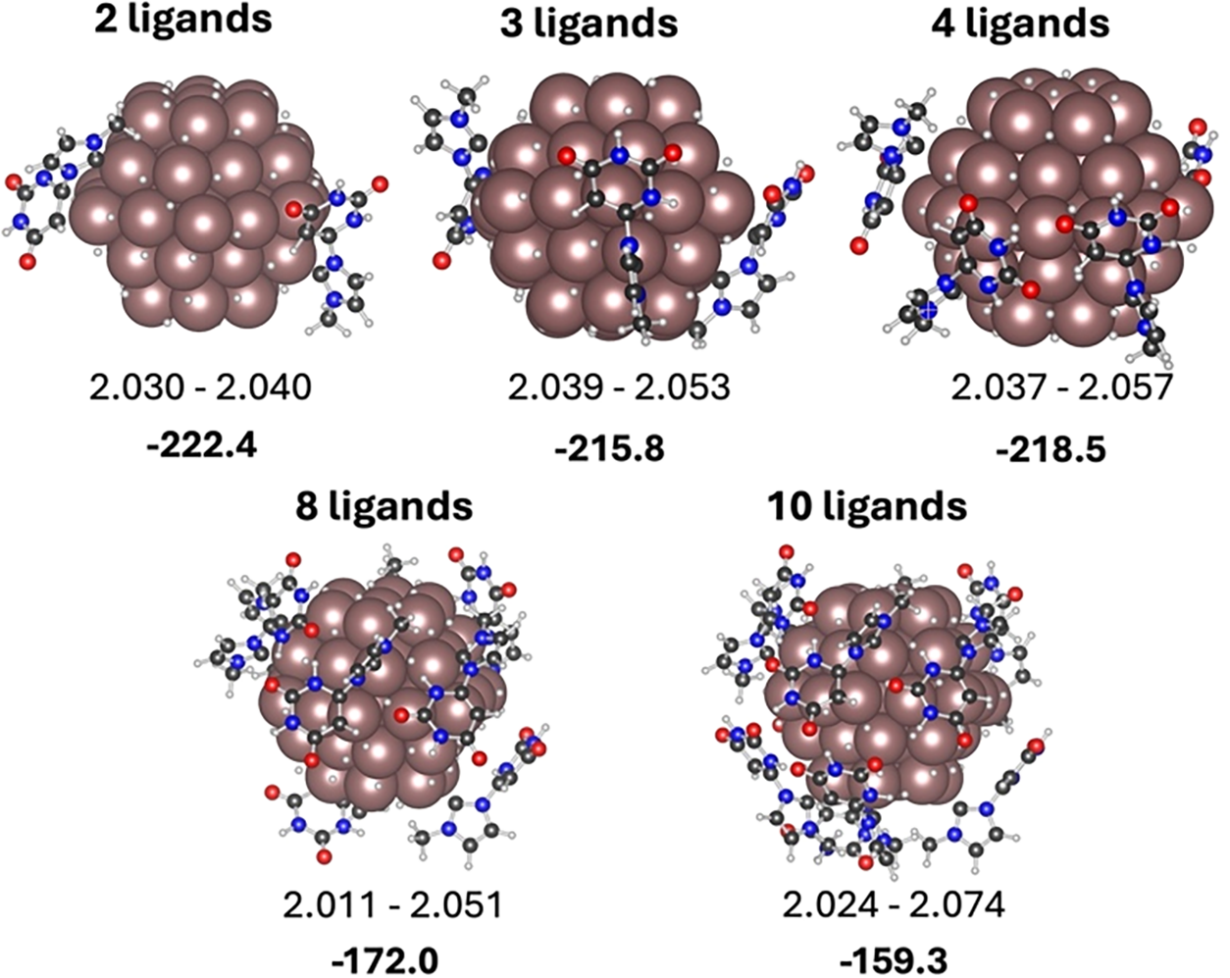
Optimized structures for the adsorption of several neutral ligands on Ru_57_H_61_. Ru-Ligand distances range in Å and relative energies with respect to the Ru_57_H_61_ NP and the isolated zwitterionic form of the ligand in kJ mol^–1^.

The mean adsorption energy per ligand when considering two or three ligands on the nanoparticle in any of the three forms is very similar to the adsorption energy of the first ligand. This is because the additional ligands are far apart and their adsorption is not influenced by the presence of the other ligands. In contrast, the addition of the fourth ligand implies a decrease on the mean adsorption energy. Indeed, diminution of the mean adsorption energy is negligible for the neutral form and small for the zwitterionic species. However, the decrease is pronounced for the anionic form. This seems related to two factors. On one side, the anionic form adsorbs in a tricoordinated mode and each adsorbed molecule blocks a larger number of active sites. On the other hand, the addition of H on the nanoparticle becomes less favorable when increasing the number of H adsorbed on the nanoparticle, enhancing the preference for the neutral form with four ligands. Increasing the number of neutral ligands adsorbed on the nanoparticle to 8 and 10 has a small effect on the mean adsorption energy, suggesting that the neutral form becomes even more preferred when increasing the ligand coverage, probably because this form only coordinates to one single site. This, again, agrees with the XPS interpretation, giving further support to **NHC-ura** as the most plausible structure on the nanoparticle.

### Antimicrobial Activity

As mentioned in the introduction, MNPs effectively target pathogens and are less prone to induce bacterial resistance compared to traditional antibiotics.^41–45^ This, together with the possibility to enhance their antibacterial performance with suitable surface ligands,^46,47^ such as uracil derivatives, presents **Ru@ura-NHC** and **Ru@ura-NHC**_**(hept)**_ as potential antibacterial drugs. **Ru@ura-NHC** and **Ru@ura-NHC**_**(hept)**_ were then tested for their antimicrobial activity against *E. coli* and *S. aureus*. For comparative purposes, the activity of the ligand **ura-zwt**, Ru NPs stabilized with a classic NHC, such as IMes (**Ru@IMes**), and the organometallic compound **Ru­(p-Cym)­ura-zwtCl** (Figure 6), were also examined.

**Figure 6:**
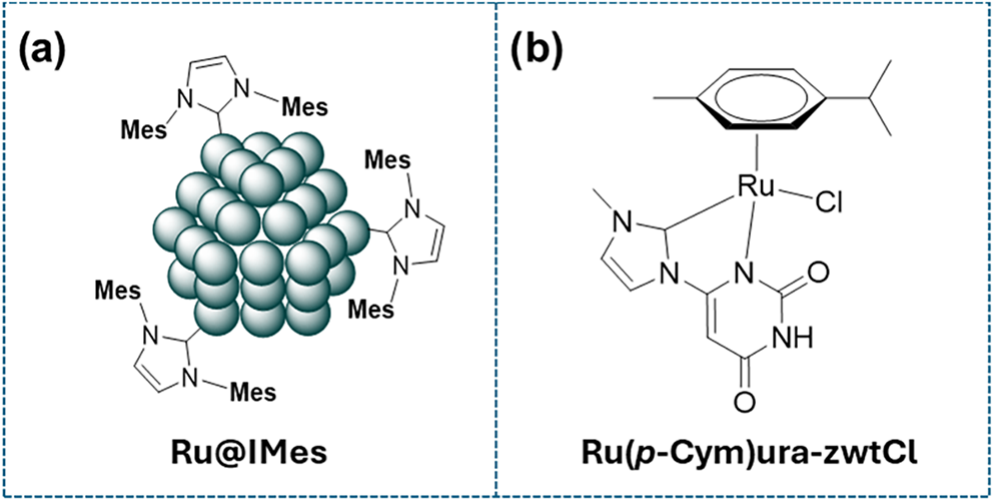
(a) Ru NPs stabilized with IMes (**RuIMes**) and (b) organometallic complex **Ru­(p-Cym) ura-zwtCl**.

As can be observed from Figure 7, **Ru@ura-NHC** showed selective antimicrobial activity against the Gram-positive pathogen *S. aureus*, while **Ru@ura-NHC**_**(hept)**_ exhibited reduced or no cytotoxic activity. On the other hand, no cytotoxic activity was observed for **Ru@ura-NHC** and **Ru@ura-NHC**_**(hept)**_ in the assay against *E. coli*. The selectivity of **Ru@ura-NHC** in the antimicrobial activity against *S. aureus* may be advantageous by minimizing off-target effects (i.e., sparing beneficial microbes) and mitigating selective pressure for resistance. This targeted activity profile positions **Ru@ura-NHC** as a promising narrow-spectrum antimicrobial candidate. Regarding the control experiments, **ura-zwt**, **Ru@IMes** (Figure 6a), and the organometallic complex (**Ru­(p-Cym)­ura-zwtCl;** (Figure 6b), none showed any antimicrobial activity (see SI, Section S.8.). This supports the synergistic role of the **ura-NHC** ligand with the Ru NPs. Seemingly, this synergy depends strongly on Ru(s)/ligand ratio. As shown in Table S1 (see SI, Section S.2.), **Ru@ura-NHC** and **Ru@ura-NHC**_**(hept)**_ exhibit significant differences in the estimated Ru(s)/ligand ratios. The **Ru@ura-NHC** shows a larger Ru(s)/ligand ratio, which might facilitate antimicrobial activity. The low Ru(s)/ligand ratio in **Ru@ura-NHC**_**(hept)**_ indicates an excess of ligand in a second coordination sphere, likely bound via base-pairing interactions, which may limit access to the Ru surface, affecting antimicrobial activity.

**Figure 7:**
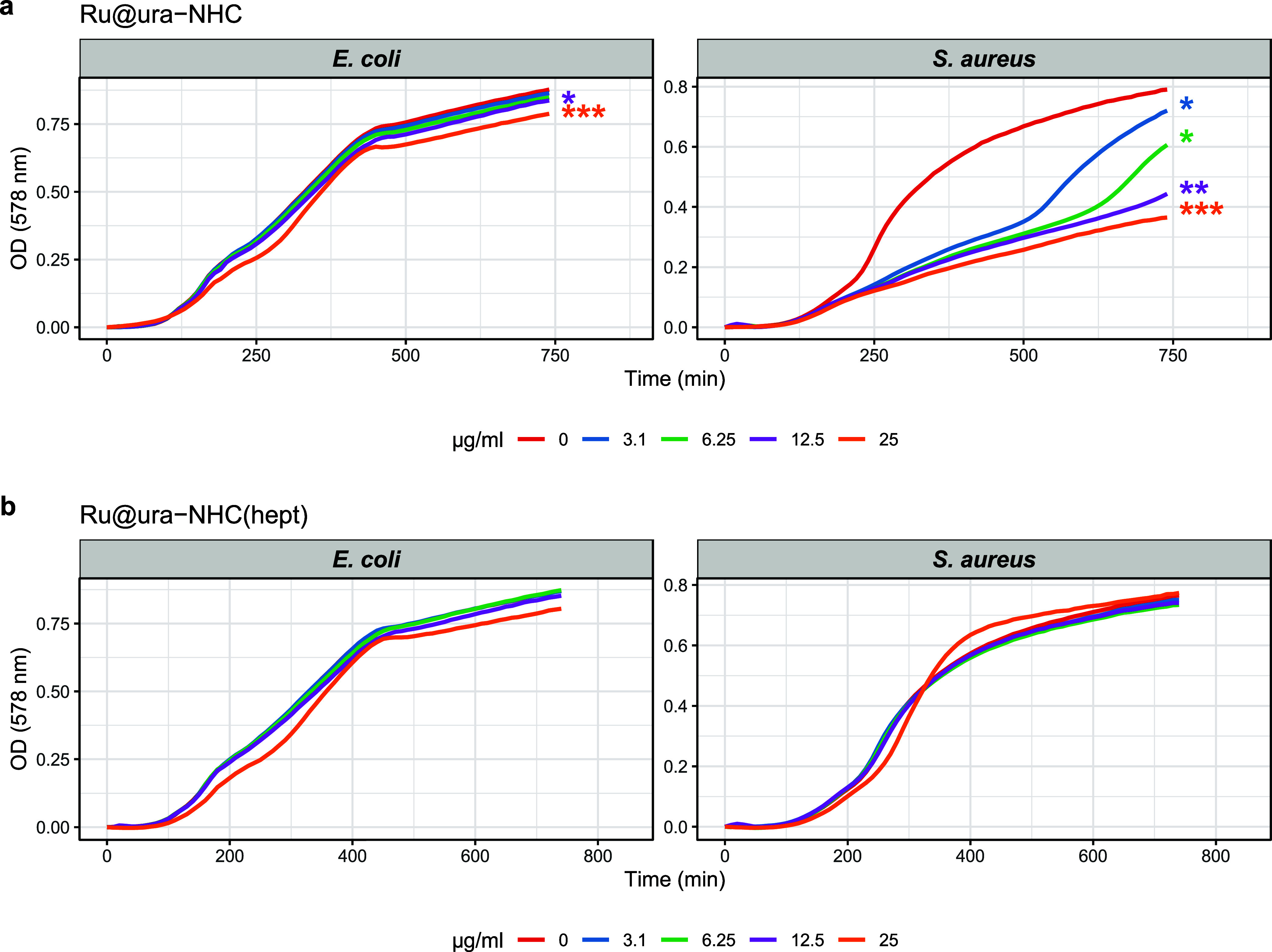
Antimicrobial activity of a) Ru@ura-NHC and (b) Ru@ura-NHC_(hept)_. Growth curves of *E. coli* and *S. aureus* treated with varying concentrations (0–25 μg/mL) of compounds. Data points represent the mean OD_578_ and error bars indicate the standard deviation (*n* = 3). Asterisks at the end of the curves denote statistically significant differences between the treated groups and the untreated control, as determined by a one-way ANOVA followed by Dunnett’s posthoc test. Significance levels are indicated as follows: *p* < 0.05 (*), *p* < 0.01 (**), and *p* < 0.001 (***); *n* = 3.

## Conclusions

Natural-based ligand-stabilized MNPs are potential antimicrobial agents due to their nonspecific toxicity, enhanced stability and targeting abilities, together with a reduced bacterial resistance. It is reported for the first time the successful application of an air-stable zwitterionic uracil-derived betaine (**ura-zwt**) as a biomimetic NHC precursor for the stabilization of Ru nanoparticles (**Ru@ura-NHC**). The mesomeric nature of **ura-zwt** enabled its use as an NHC source for nanoparticle stabilization with 100% atom economy, preventing byproduct generation. The Ru NPs obtained were characterized using TEM, HRTEM and ICP-OES. The coordination of the uracil-based NHC to the Ru surface was investigated through a combined theoretical/experimental study. FT-IR, MAS NMR and XPS spectroscopies confirmed the presence and coordination mode of the ligand at the Ru surface. DFT calculations indicated that the coordination of the neutral form (**ura-NHC**) is preferred over the zwitterionic form (**ura-zwt**), especially at high ligand coverage. **Ru@ura-NHC** was applied as an antimicrobial agent against *E. coli* and *S. aureus*. These Ru NPs demonstrated selective antimicrobial activity against the Gram-positive pathogen *S. aureus*, while the larger nanoparticles stabilized with the same uracil-derived NHC (**Ru@ura-NHC**_**(hept)**_) showed reduced cytotoxic activity. Control experiments using the free ligand (**ura-zwt)**, Ru NPs stabilized with nonfunctionalized NHCs (**Ru@IMes**), and a **ura-NHC**-stabilized organometallic complex (**Ru­(p-Cym)­(NHC)­Cl**) did not show any antimicrobial activity. This supports the idea that the synergy between the **ura-NHC** ligand and the Ru NPs is crucial for antimicrobial effectiveness. The enhanced activity of **Ru@ura-NHC** compared to **Ru@ura-NHC**_**(hept)**_ can be attributed to a higher surface-area-to-volume ratio and lower surface coverage, which may facilitate the penetration of the Ru NPs through bacterial cells. The selective antibacterial activity was successfully demonstrated, highlighting the potential of these hybrid organic–inorganic nanosystems in combating pathogens. For **Ru@ura-NHC**, antimicrobial activity is observed at concentrations as low as 3.1 μg/mL, a low value when compared to those reported for other nanoparticles.^73^ The use of natural ligands to stabilize MNPs not only paves the way for antimicrobial applications but also offers the possibility to utilize them as recognition units for biological systems, which will be the focus of our future work.

## Supplementary Material



## Notes

Additional experimental and computational details (PDF)
