# Supplementary material for: Ru Nanoparticles Ligated by an N‑Heterocyclic Carbene Derived from Uracil Nucleoside as Selective Antimicrobial Agents
Source: Inorg Chem. 2026 Feb 16;65(8):4580–91. doi: 10.1021/acs.inorgchem.5c05649 (PMC12958292; doi:10.1021/acs.inorgchem.5c05649)
Supplement: Supplementary file 1 [file ic5c05649_si_001.pdf]

Supporting Information

Ru Nanoparticles Ligated by an N-heterocyclic Carbene Derived from Uracil Nucleoside as Selective Antimicrobial Agents

Adrián Sánchez,[a] Luis A. M. Carrascosa,[a] Giulia Romeo, [b] Giulia Orsini, [b] Yannick Coppel,[c] Sarela Santamarina,[d] Luis Rodríguez-Santiago,[e] Xavier Solans-Monfort,[e] Ana Petronilho,[b], Luis M. Martínez-Prieto[a],**

[a] IIQ, Instituto de Investigaciones Químicas (CSIC-Universidad de Sevilla), Consejo Superior de Investigaciones Científicas, Avda. Americo Vespucio 49, 41092 Seville, Spain

[b] *Instituto de Tecnologia Química e Biológica António Xavier, Av. da Republica, 2780-157 Oeiras, Portugal*

[c] CNRS, LCC (Laboratoire de Chimie de Coordination), Université de Toulouse, UPS, INPT, 205 route de Narbonne, BP 44099, F-31077 Toulouse Cedex 4, France

[d] Department of Deep Microbiome Metabolomics, Leibniz Institute for Natural Product Research and Infection Biology (Leibniz-HKI), Beutenbergstrasse 11a, 07745, Jena, Germany

[e] Departament de Química, Universitat Autònoma de Barcelona, 08193 Cerdanyola del Vallès (Barcelona), Spain

Emails: ana.petronilho@itqb.unl.pt; luismiguel.martínez@csic.es

Table of Contents

S.1. Abbreviations 2

S.2. ICP-OES 3

S.3. Base Pairing ura-zwt 3

S.4. FT-IR 9

S.5. MAS-NMR 10

S.6. XPS 10

S.7. DFT Calculations 11

S.8. Antimicrobial tests 20

S.1. Abbreviations

ATR. Attenuated total reflectance.

DMF-$_{d7}$. Deuterated dimethylformamide.

DMSO-$_{d6}$. Deuterated dimethylsulfoxide.

FT-IR. Fourier-Transformed Infrared spectroscopy.

hcp. Hexagonal Close-Packed.

ICP-OES. Inductively Coupled Plasma-Optical Emission Spectrometry.

LB. Lysogeny Broth.

MAS-NMR. Magic Angle Spinning NMR.

MNPs. Metal nanoparticles.

NHC. N-Heterocyclic Carbene.

NMR. Nuclear magnetic resonance.

NPs. Nanoparticles.

OD. Optical density.

PAW. Projector Augmented Wave method.

***p*-Cym.** *p*-Cymene.

PBE. Perdew-Burke-Ernzerhof.

Ru(COD)(COT). 1,5-Cyclooctdiene(1,3,5-cyclooctatriene)ruthenium(0).

Ru@hept. Ru nanoparticles stabilized by *n*-heptanol.

Ru@ura-NHC. Ru nanoparticles stabilized by **ura-NHC** ligand.

Ru@ura-NHC$_{(hept)}$. Ru nanoparticles stabilized by **ura-NHC** obtained after a ligand exchange process of **Ru@hept** with **ura-zwt**.

TEM/HRTEM. Transmission electron microscopy/high-resolution transmission electron microscopy.

THF. Tetrahydrofuran.

TMS. Tetramethylsilane.

ura-zwt. 6-(3-Methylimidazolio)-2,4(3H)-pyrimidinedionate.

ura-NHC. 6-(3-Methylimidazolin-2-ylidene)-2,4(1H,3H)-pyrimidinedionate.

VASP. Vienna Ab initio Simulation Package.

XPS. X-ray photoelectron spectroscopy.

S.2. ICP-OES

Table S1. *Theoretical composition of* ***Ru@ura-NHC*** *and* ***Ru@ura-NHC$_{(hept)}$****.*

MNPs[a]	Size (nm)	% Ru[a]	Ru/L Ratio	Ru/L[b]	Ru(s)[c]	Ru(s)/L_y
**Ru@ura-NHC**	2.1 ± 02	47.5	1.7:1	381/224	189	0.84
**Ru@ura-NHC$_{(hept)}$**	3.2 ± 05	40.5	1.3:1	1376/1058	482	0.46

[a] % of Ru obtained by Inductively Coupled Plasma Optical Emission spectroscopy (ICP-OES). [b] The approximate composition is based on the Ru/L ratio and the mean diameter measured by TEM.[c] Number of surface atoms. Approximate values obtained from *ChemCatChem* **2011**, 3, 1413-1418.

S.3. Base Pairing ura-zwt

S.3.1. Self-base pairing: The variations in the chemical shifts of the N3-H group of **ura-zwt** were monitored by ^{1}H NMR to determine the formation of the corresponding base pair (Figure S1). Two samples were prepared in DMSO-d_6, with a concentration of 20 mM of **ura-zwt** (a) and 100 mM (b), respectively, and the ^{1}H NMR spectra of both solutions were recorded (Figure S2).

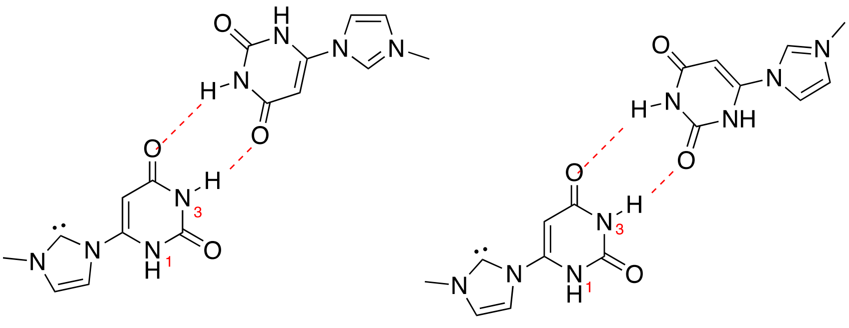

I II

Figure S1. *Self-base pairing of uracil ligands.*

S.3.2. RuNP:ura-zwt: The variations in the chemical shifts of the N3-H group of **ura-zwt** in the presence of nanoparticles were monitored by ^{1}H NMR to determine the possible interaction between the coated **RuNPs** and **ura-zwt.** Two samples of 20 mM of **ura-zwt** in DMSO-d_6 were prepared. 10 mg of **Ru@ura-NHC** were added to the first sample and 10 mg of **Ru@ura-NHC$_{(hept)}$** were added to the second sample and the ^{1}H NMR spectra of both solutions were recorded (**Figures S3-S4**). The experiment was repeated under the same conditions using DMF-d_7 as solvent (**Figures S5-S6**).

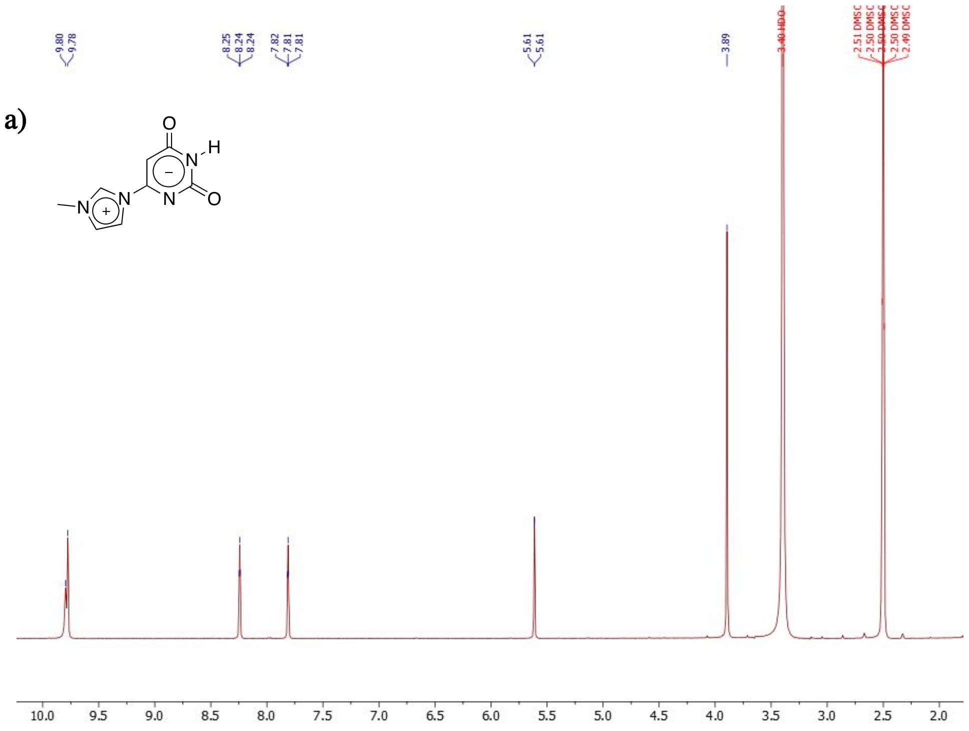

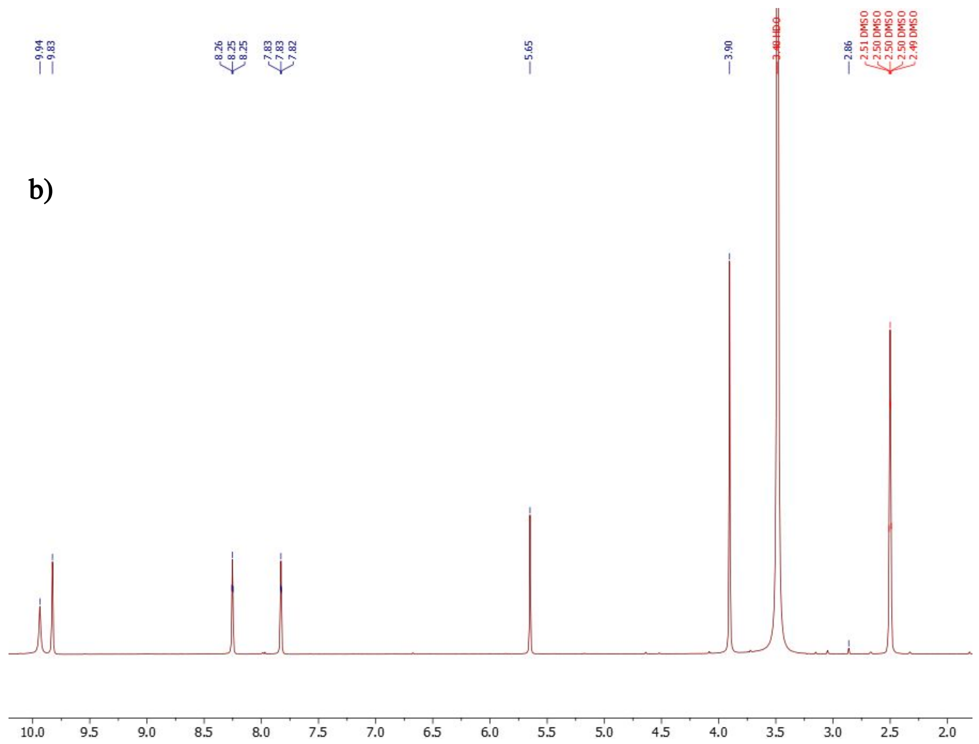

Figure S2. ***a)*** *^{1}H NMR spectrum of the compound recorded in DMSO-d_6 at a concentration of 20 mM of* **ura-zwt** *in DMSO-d_6.* ***b)*** *^{1}H NMR spectrum of the compound recorded in DMSO-d_6 at a concentration of 100 mM of* **ura-zwt** *in DMSO-d_6.*

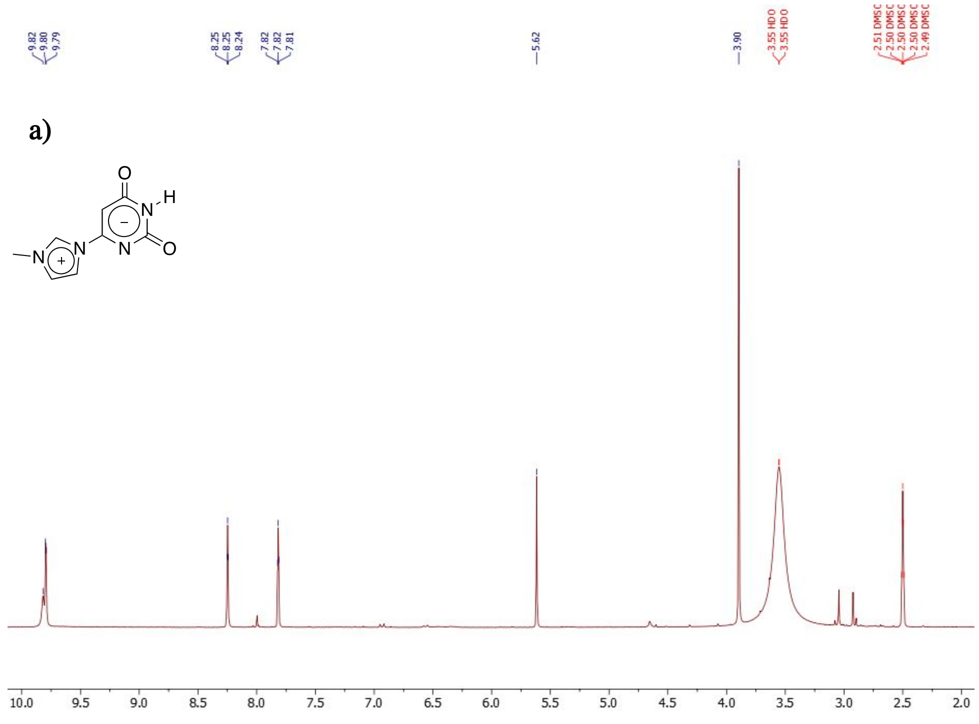

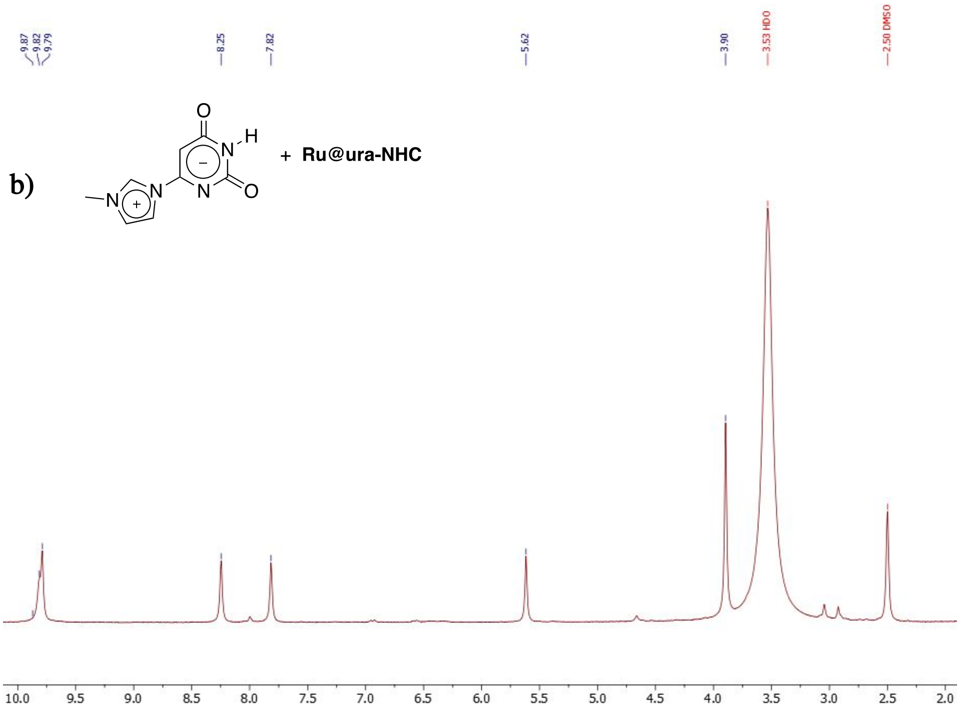

Figure S3. ***a)*** *^{1}H NMR spectrum of* **ura-zwt** *recorded at a concentration of 20 mM in DMSO-d6.* ***b)*** *^{1}H NMR spectrum of the* **ura-zwt** *recorded in DMSO-d_6 at a concentration of 20 mM after the addition of 10 mg of* ***Ru@ura-NHC.***

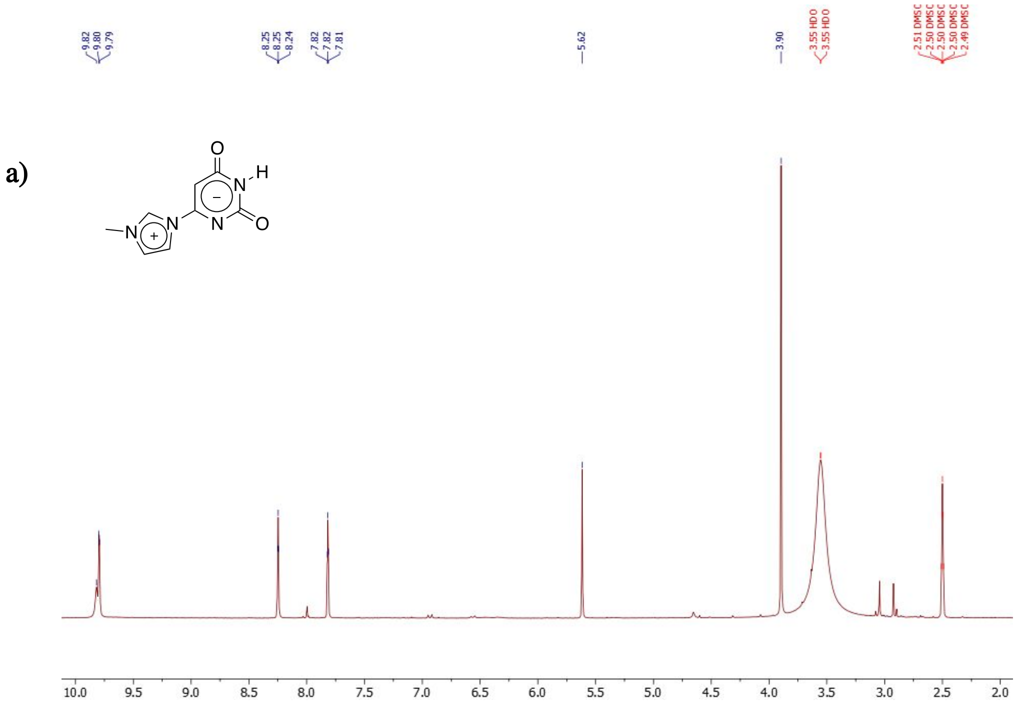

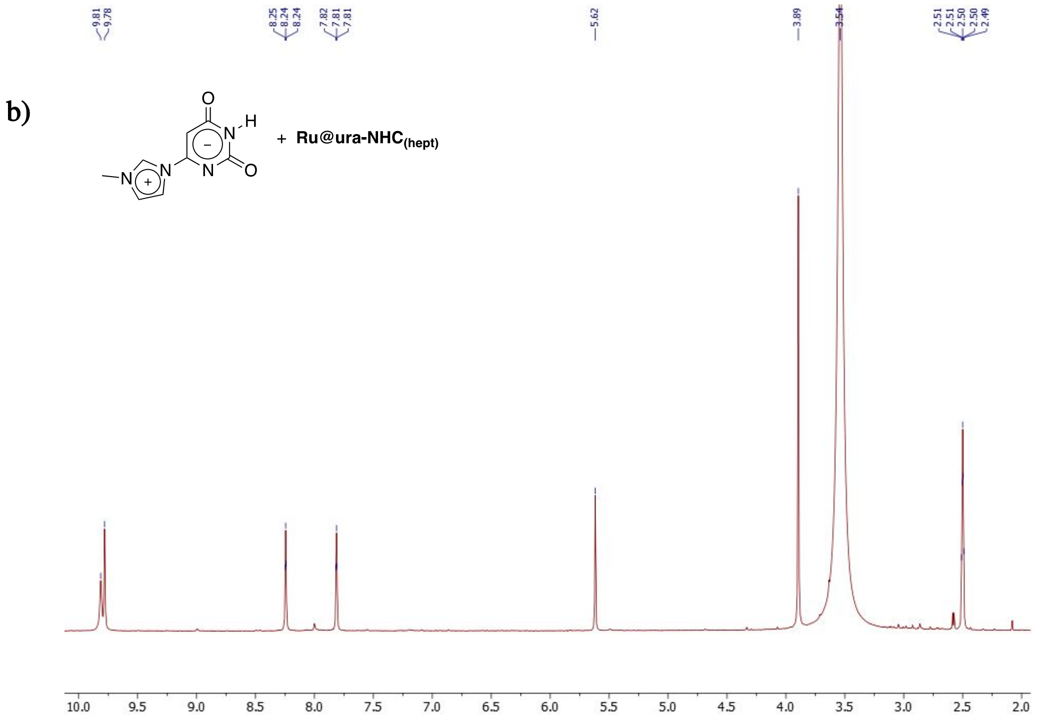

Figure S4. ***a)*** *^{1}H NMR spectrum of* **ura-zwt** *recorded at a concentration of 20 mM of* **ura-zwt** *in DMSO*-d_6. ***b)*** *^{1}H NMR spectrum of the compound recorded in DMSO-d_6 at a concentration of 20 mM of* **ura-zwt** *after the addition of 10 mg of* ***Ru@ura-NHC$_{(hept)}$.***

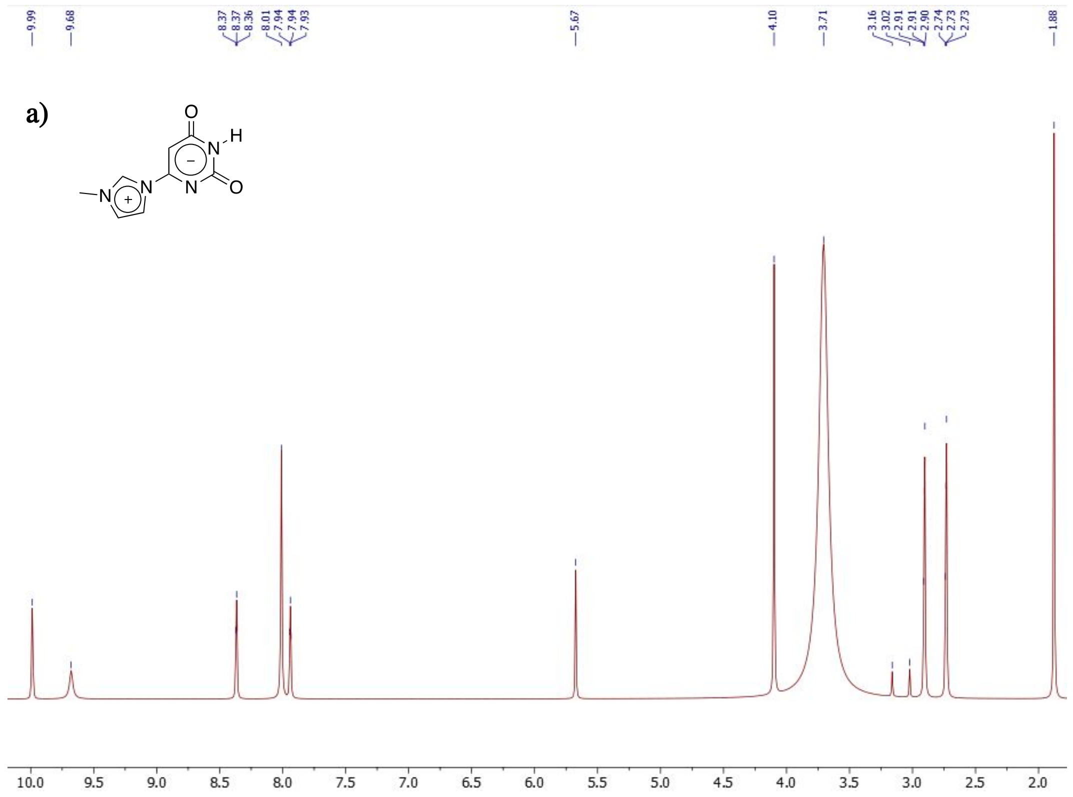

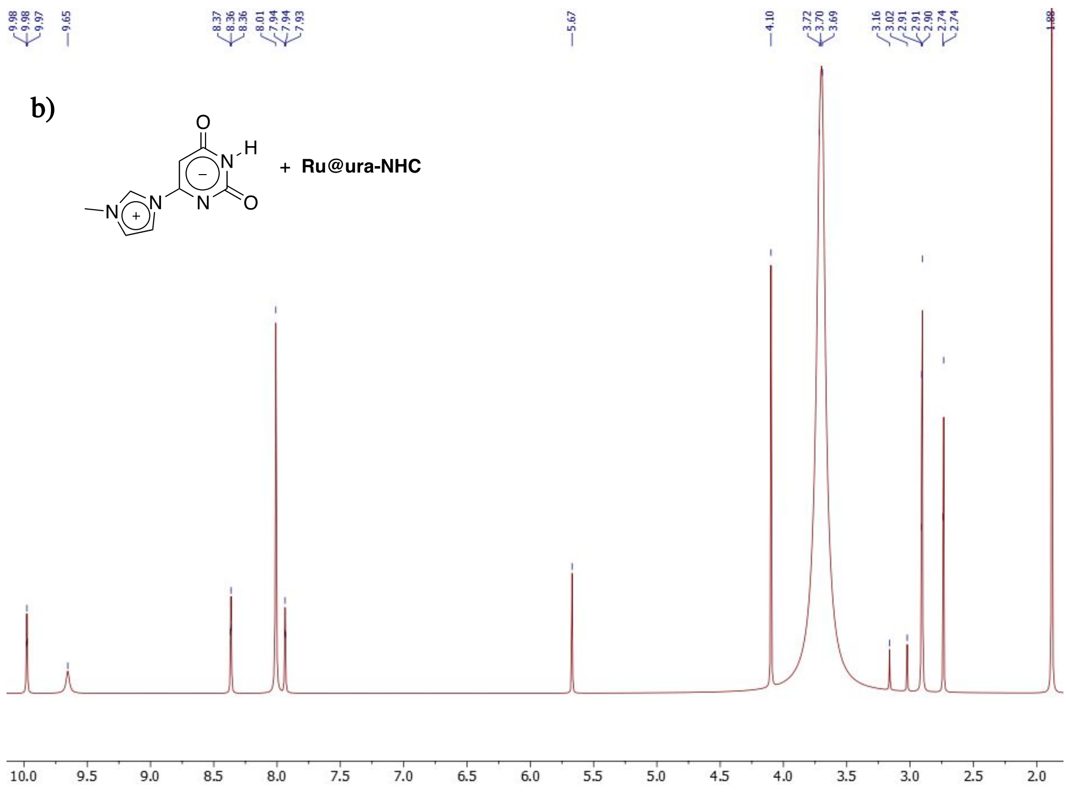

Figure S5. ***A)*** *^{1}H NMR spectrum of the compound recorded at a concentration of 20 mM of* **ura-zwt** *in DMF-d₇.* ***B)*** *^{1}H NMR spectrum of the compound recorded at a concentration of 20 mM of* **ura-zwt** *in DMF-d₇ after the addition of 10 mg of* ***Ru@ura-NHC***.

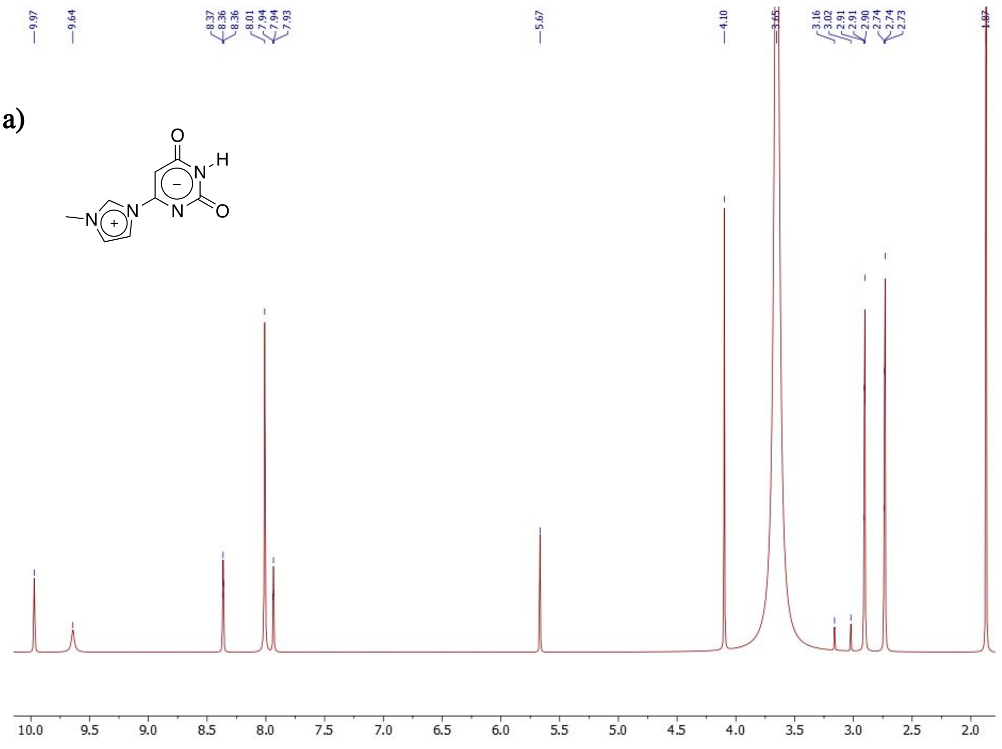

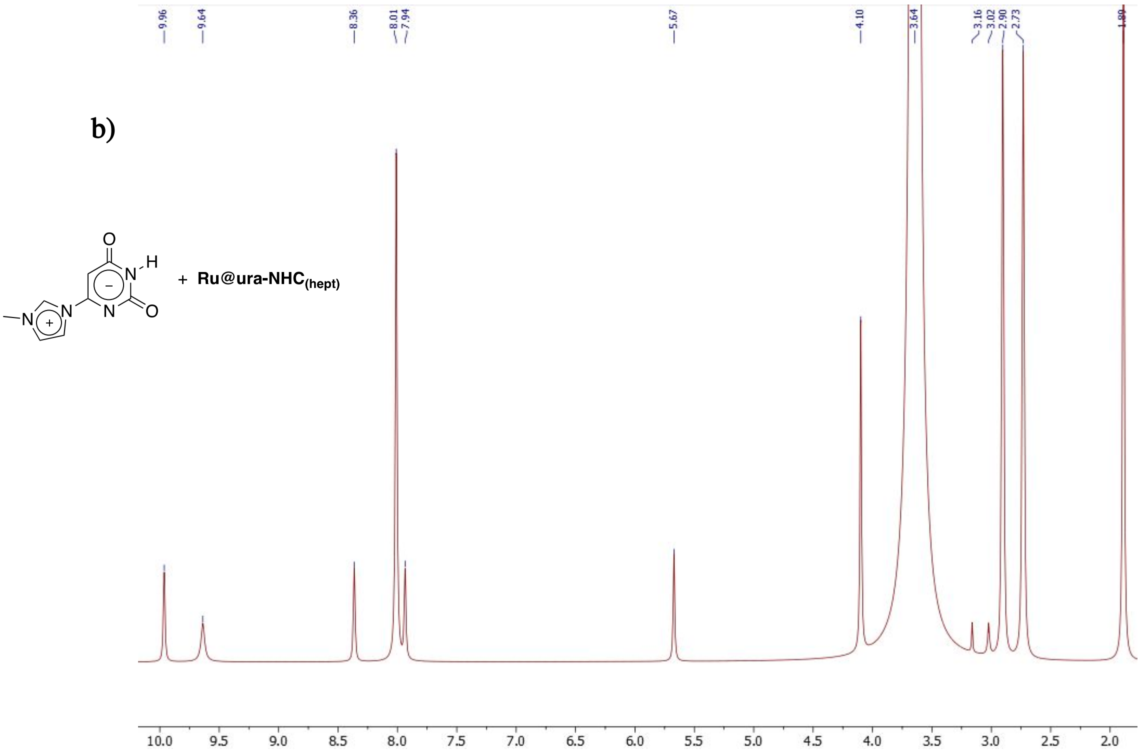

Figure S6. ***a)*** *^{1}H NMR spectrum of the compound recorded at a concentration of 20 mM of* **ura-zwt** *in DMF-d$_7$.* ***b)*** *^{1}H NMR spectrum of the compound recorded at a concentration of 20 mM of* **ura-zwt** *in DMF-d$_7$ after the addition of 10 mg of* ***Ru@ura-NHC$_{(hept)}$.***

S.4. FT-IR

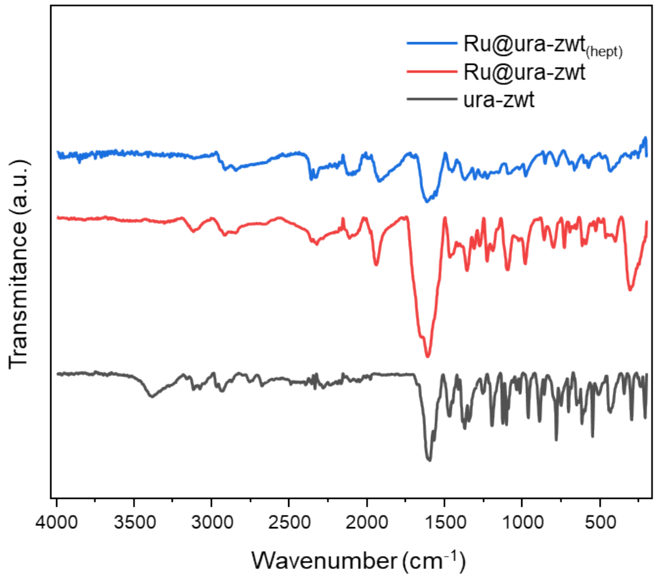

Figure S7. *ATR FT-IR spectra of* ***a) ura-zwt*** *(grey),* ***b) Ru@ura-NHC*** *(red) and* ***c) Ru@ura-NHC$_{(hept)}$****(blue).*

S.5. MAS-NMR

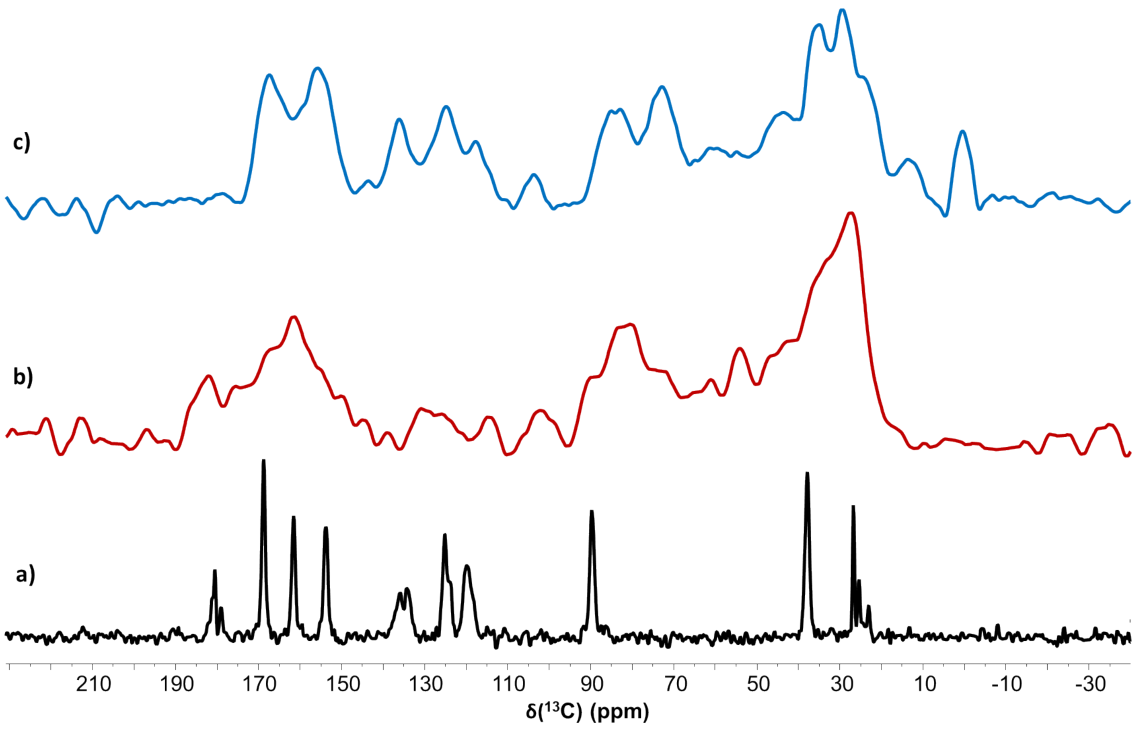

Figure S8. *^{13}C CP-MAS-Hahn NMR spectra of* ***a) ura-zwt*** *(grey),* ***b) Ru@ura-NHC*** *(red) and* ***c) Ru@ura-NHC$_{(hept)}$****(blue).*

S.6. XPS

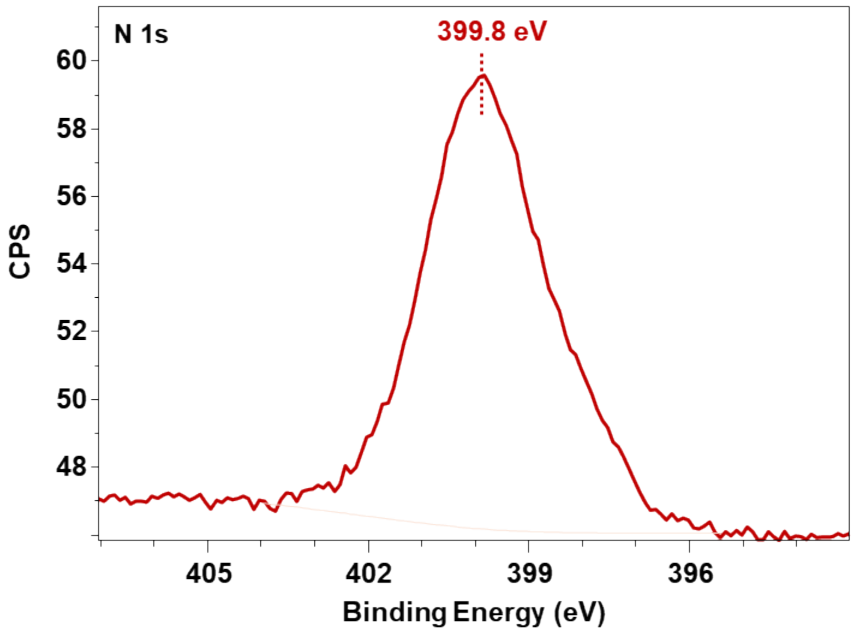

Figure S9. *X-ray photoelectron spectroscopy (XPS) of the N 1s signal of* ***Ru@ura-NHC$_{(hept)}$****.*

S.7. DFT Calculations

Two different models were used in the DFT calculations to better understand the interaction between the uracil-bearing NHC ligand and the Ru-based nanoparticle: i) a (100) hpc ruthenium slab model derived from previous contributions of some of us and consisting on a $5x2$ $\sqrt{}(2R\_(30°)$ supercell that contains 20 Ru atoms on the outermost layer;[1] ii) the $Ru_{57}H_{61}$ nanoparticle employed before by us for analyzing the catalytic activity of small Ru-nanoparticles on hydrogen evolution reaction.[2] This nanoparticle presents an hpc structure and mainly shows exposed Ru centers from the (100) and (101) facets.

All calculations were performed with the PBE[3] density functional as implemented in VASP. [4,5] Dispersion forces are considered by adding Grimme's D3 empirical correction.[6] Atomic cores are described with PAW pseudopotentials[7,8] and the valence electrons are represented with a plane-wave basis set with an energy cutoff of 500 eV. Moreover, solvent effects were included by performing single-point calculations at the gas-phase optimized structure using the continuum

---

[1] Fenoll, D. A.; Sodupe, M.; Solans-Monfort, X., Different role of ruthenium and platinum defective sites on the catalytic activity for the hydrogen evolution reaction. *Catal. Today* **2024,** *442*, 114908. https://doi.org/10.1016/j.cattod.2024.114908; https://doi.org/10.1039/D3QI00698K.

[2] Hou, H.; Cerezo-Navarrete, C.; Fenoll, D. A.; Kraft, M.; Marini, C.; Rodríguez-Santiago, L.; Solans-Monfort, X.; Martínez-Prieto, L. M.; Romero, N.; García-Antón, J.; Sala, X., Enhanced electrocatalytic hydrogen evolution with bimetallic Ru/Pt nanoparticles supported on nitrogen-doped reduced graphene oxide. *Inorg. Chem. Front.* **2025,** *12* (15), 4569-4582. https://doi.org/10.1039/D5QI00451A.

[3] Perdew, J. P.; Burke, K.; Ernzerhof, M., Generalized Gradient Approximation Made Simple. *Phys. Rev. Lett.* **1996,** *77* (18), 3865-3868. https://doi.org/10.1103/PhysRevLett.77.3865.

[4] Kresse, G.; Hafner, J., Ab initio molecular dynamics for liquid metals. *Physical Review B* **1993,** *47* (1), 558-561. https://doi.org/10.1103/PhysRevB.47.558; b) Kresse, G.; Furthmüller, J., Efficient iterative schemes for ab initio total-energy calculations using a plane-wave basis set. *Phys. Rev. B* **1996,** *54* (16), 11169-11186. https://doi.org/10.1103/PhysRevB.54.11169.

[5] Kresse, G.; Furthmüller, J., Efficient iterative schemes for ab initio total-energy calculations using a plane-wave basis set. *Phys. Rev. B* **1996,** *54* (16), 11169-11186. https://doi.org/10.1103/PhysRevB.54.11169.

[6] Grimme, S.; Antony, J.; Ehrlich, S.; Krieg, H., A consistent and accurate ab initio parametrization of density functional dispersion correction (DFT-D) for the 94 elements H-Pu. *J. Chem. Phys.* **2010,** *132* (15). https://doi.org/10.1063/1.3382344.

[7] Kresse, G.; Joubert, D., From ultrasoft pseudopotentials to the projector augmented-wave method. *Phys. Rev. B* **1999,** *59* (3), 1758-1775. https://doi.org/10.1103/PhysRevB.59.1758; b) Blöchl, P. E., Projector augmented-wave method. *Phys. Rev. B* **1994,** *50* (24), 17953-17979. https://doi.org/10.1103/PhysRevB.50.17953.

[8] Blöchl, P. E., Projector augmented-wave method. *Phys. Rev. B* **1994,** *50* (24), 17953-17979. https://doi.org/10.1103/PhysRevB.50.17953.

model implemented in VASPsol and water as solvent.[9] Calculations with the nanoparticle model were performed at Γ point due to the dimensionality of the model, while those of the slab, were performed with a (2x2x1) Monkhorst mesh of points of the reciprocal lattice.

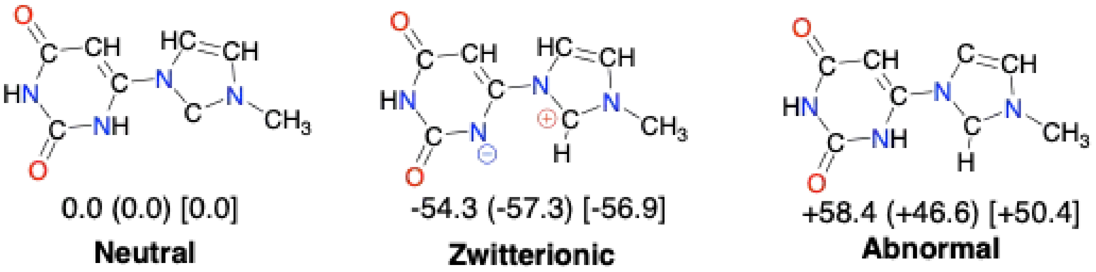

Figure S10. *Comparison between the Gaussian16 and VASP relative energies in kJ mol^{-1} of the different forms of the ligand. Legend: B3LYP/6-311+G(d,p), (PBE-D3/6-311+G(d,p) and [PBE-D3/cutoff 500eV],*

[9] Mathew, K.; Sundararaman, R.; Letchworth-Weaver, K.; Arias, T. A.; Hennig, R. G., Implicit solvation model for density-functional study of nanocrystal surfaces and reaction pathways. *The J. Chem. Phys.* **2014,** *140* (8). https://doi.org/10.1063/1.4865107.

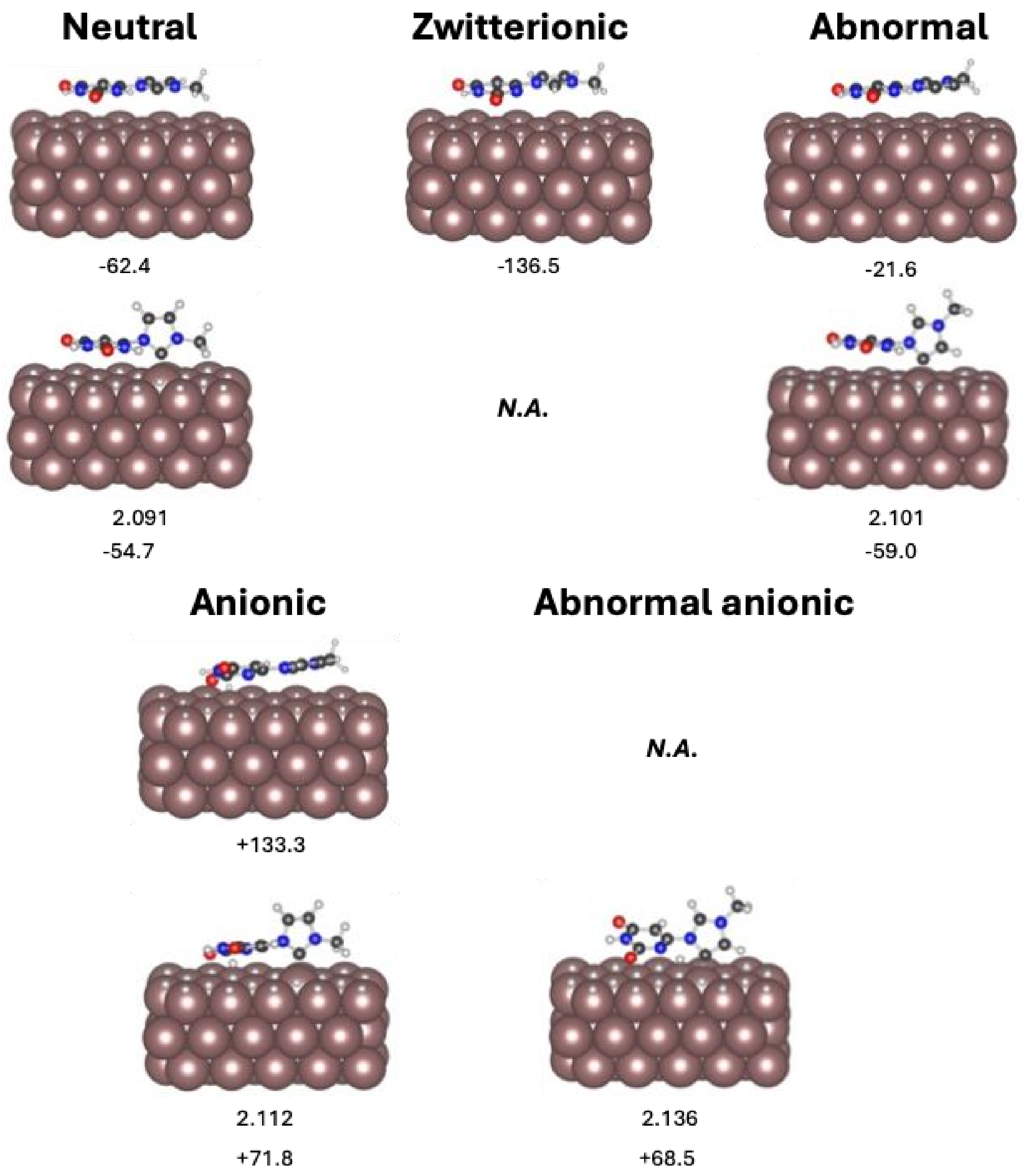

Figure S11. *Optimized structures for the adsorption of the NHC-uracil bearing ligand on the crystalline slab periodic model. Ru-L distances in Å and adsorption energies with respect to separated surface and zitterionic form in kJ mol^{-1}.*

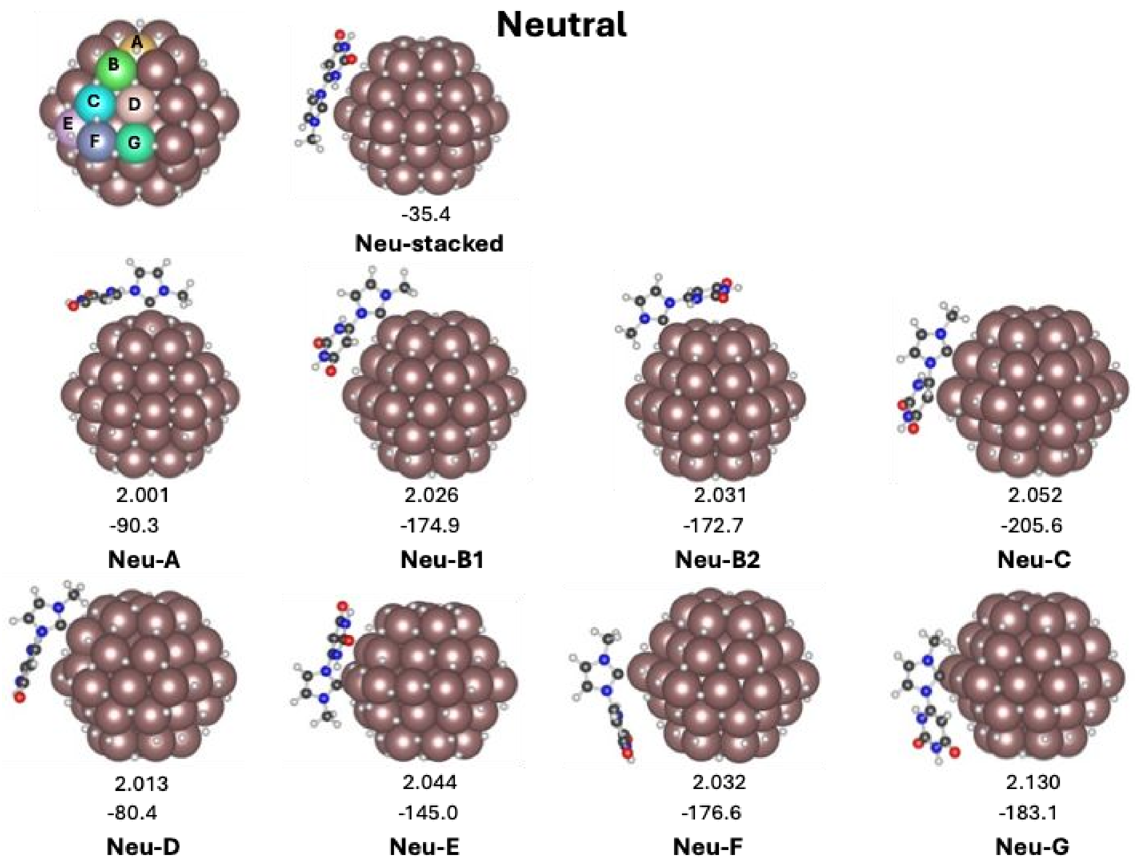

Figure S12. *Optimized structures for the adsorption of the NHC-uracil bearing ligand neutral form on the seven potential adsorption sites of* $Ru_{57}H_{61}$ *nanoparticle model. Ru-L distances in Å and adsorption energies with respect to separated surface and zitterionic form in kJ* mol^{-1}.

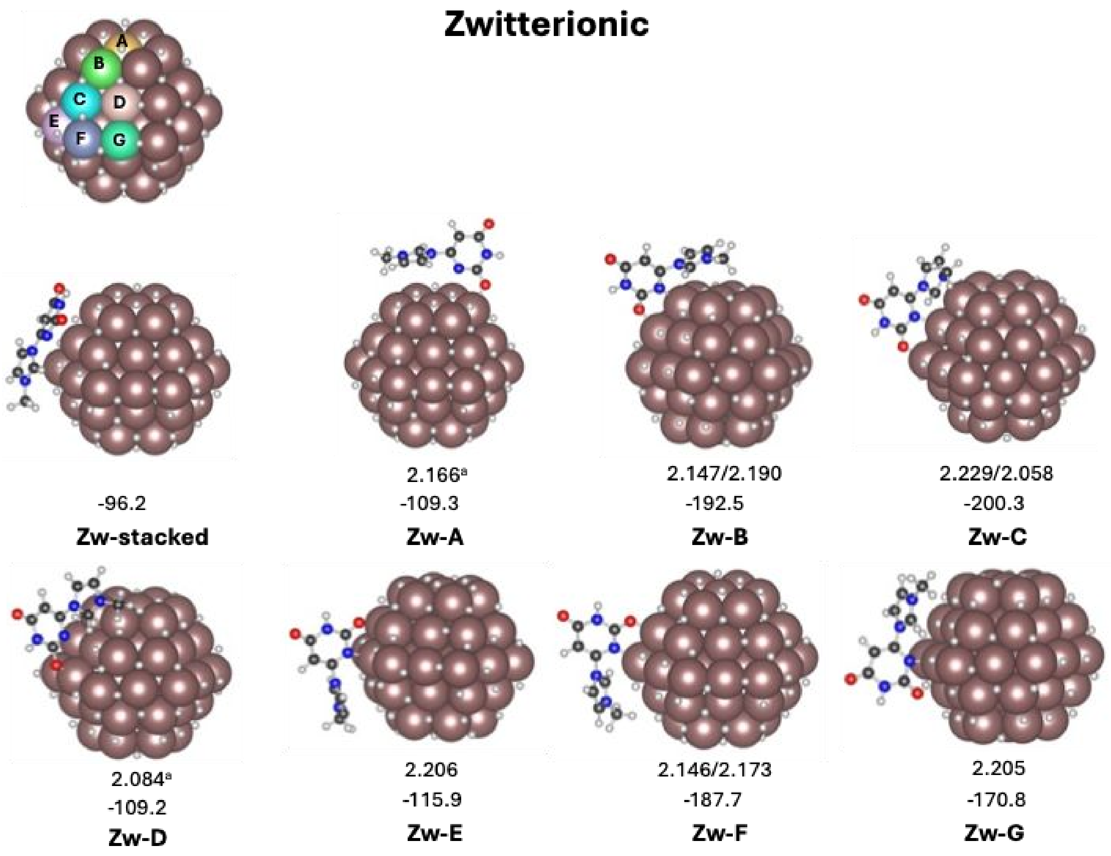

Figure S13. *Optimized structures for the adsorption of the NHC-uracil bearing ligand zwitterionic form on the seven potential adsorption sites of* $Ru_{57}H_{61}$ *nanoparticle model. Ru-L distances in Å and adsorption energies with respect to separated surface and zitterionic form in kJ* mol^{-1}.

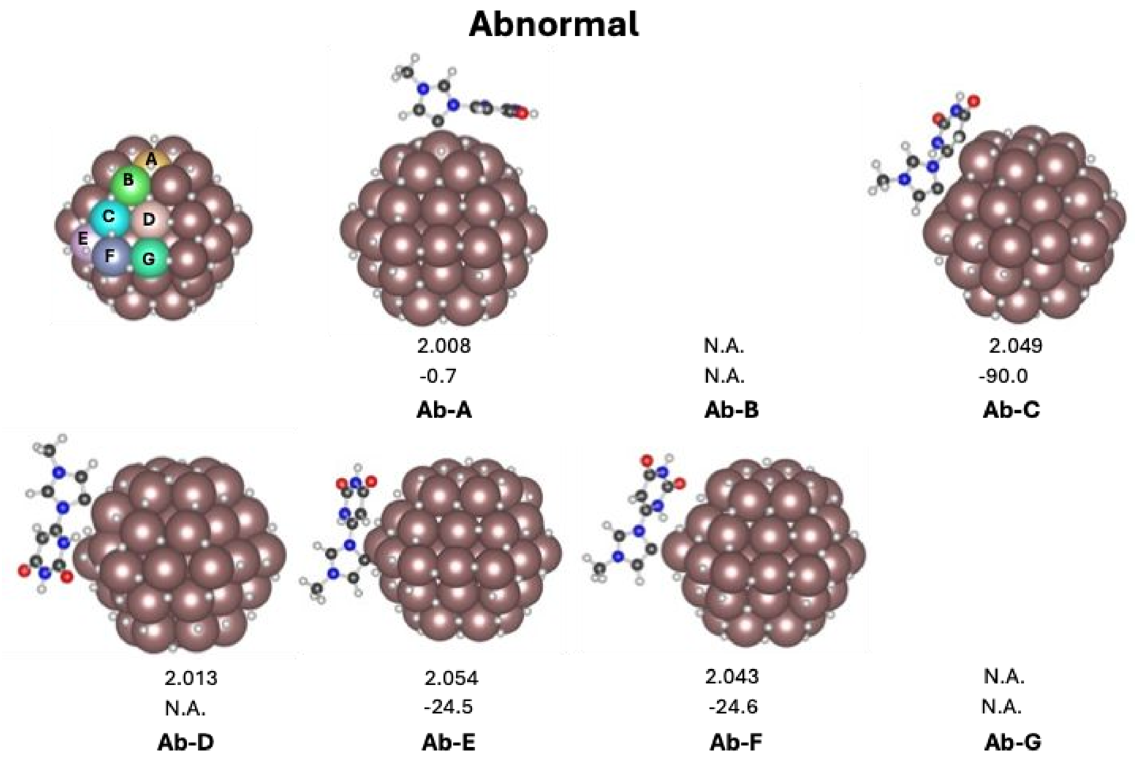

Figure S14. *Optimized structures for the adsorption of the NHC-uracil bearing ligand abnormal form on the seven potential adsorption sites of $Ru_{57}H_{61}$ nanoparticle model. Ru-L distances in Å and adsorption energies with respect to separated surface and zitterionic form in kJ mol^{-1}.*

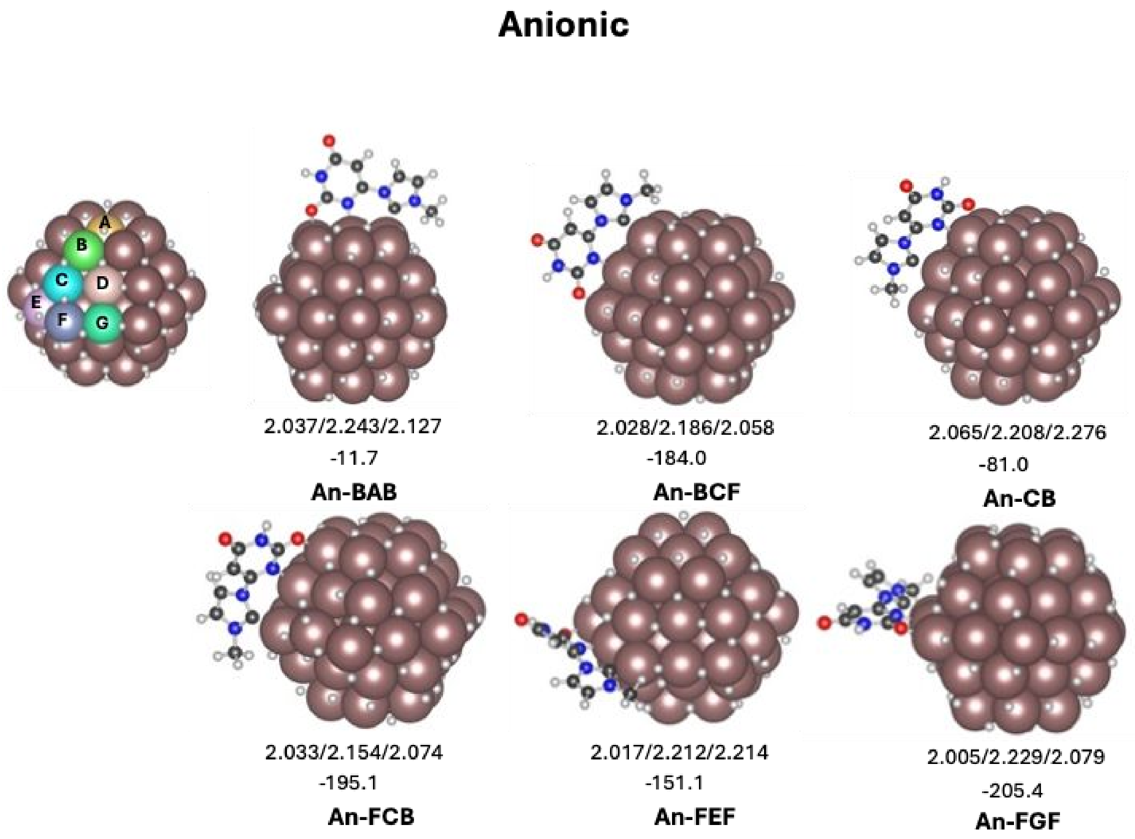

Figure S15. *Optimized structures for the adsorption of the NHC-uracil bearing ligand anionic form on several potential adsorption sites of $Ru_{57}H_{61}$ nanoparticle model. Ru-L distances in Å and adsorption energies with respect to separated surface and zitterionic form in kJ mol^{-1}.*

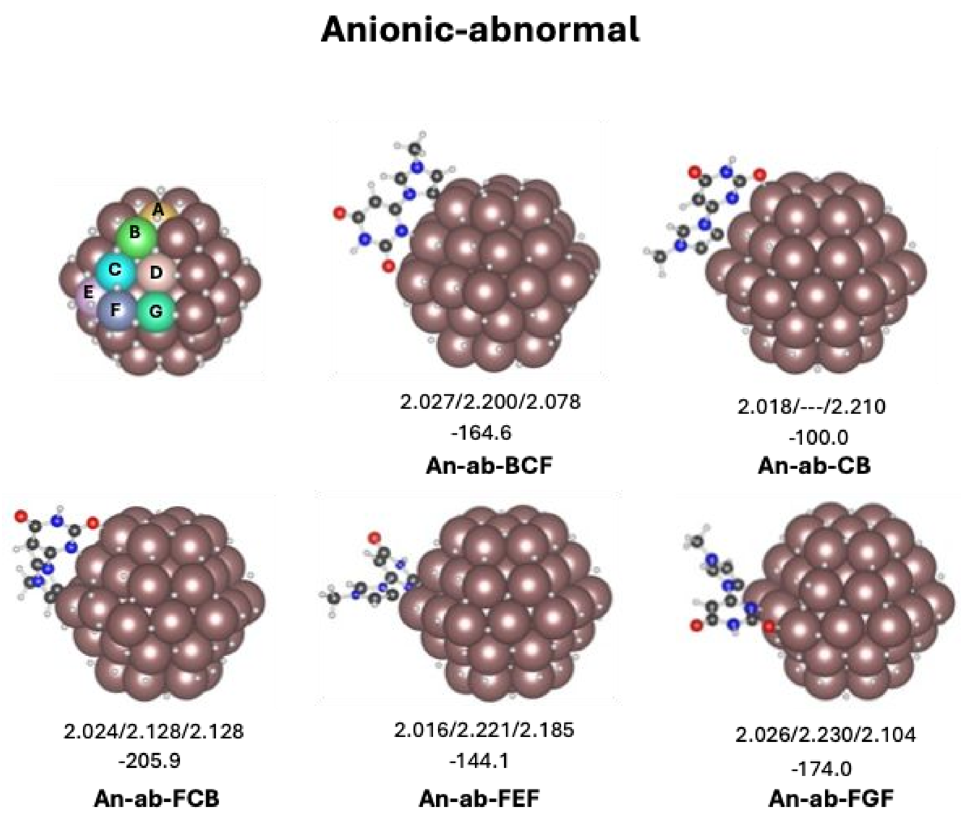

Figure S16. *Optimized structures for the adsorption of the NHC-uracil bearing ligand abnormal anionic form on several potential adsorption sites of* $Ru_{57}H_{61}$ *nanoparticle model. Ru-L distances in Å and adsorption energies with respect to separated surface and zitterionic form in kJ* mol^{-1}.

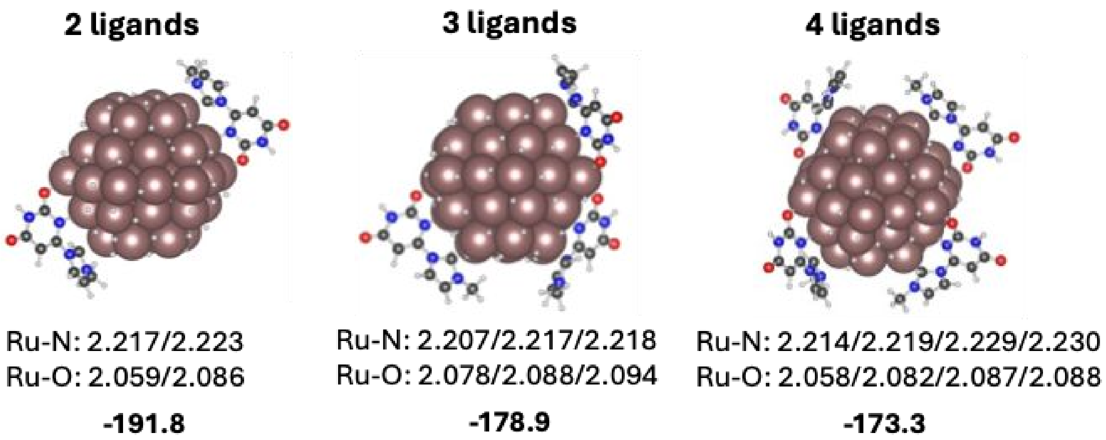

Figure S17. *Optimized structures for the adsorption of several NHC-uracil bearing ligand zwitterionic form on $Ru_{57}H_{61}$ NP model. Ru-L distances in Å and adsorption energies with respect to separated surface and zitterionic form in kJ mol^{-1}.*

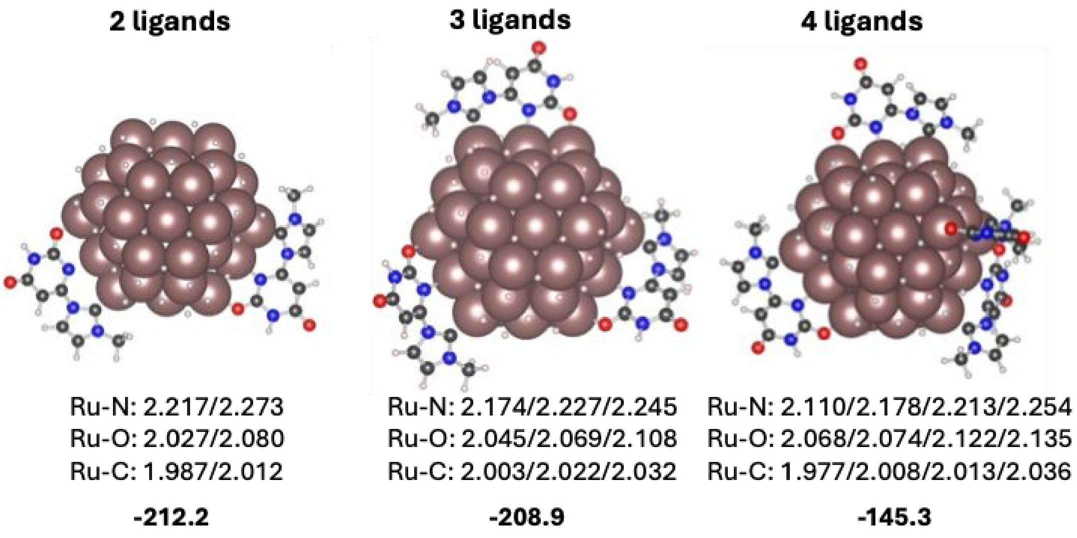

Figure S18. *Optimized structures for the adsorption of several NHC-uracil bearing ligand anionic form on $Ru_{57}H_{61}$ NP model. Ru-L distances in Å and adsorption energies with respect to separated surface and zitterionic form in kJ mol^{-1}.*

S.8. Antimicrobial tests

Strains uropathogenic *Escherichia coli* UTI89 and *Staphylococcus aureus* USA300 were streaked from frozen stocks onto Lysogeny Broth (LB) agar plates. Single colonies were inoculated into liquid LB and grown overnight at 37 °C with shaking. Overnight cultures were diluted to an optical density (OD) of 0.01 and either left untreated or treated with nanoparticles or negative controls as described below.

Nanoparticle stocks (ASH312 and ASH330) and the negative control **Ru@IMes** were prepared at 30 mg/ml in sterile water. **Ru(*p*-Cym)ura-zwtCl** was prepared at 3.75 mg/ml, and betaine **ura-zwt** at 0.0822 M (equivalent to 30 mg/ml nanoparticle concentration). Stocks were diluted in LB to final concentrations of 3.1, 6.25, 12.5, and 25 µg/ml. Bacterial cultures were incubated with these treatments in a final volume of 100 µl in 96-well plates. These were incubated at 37 °C with continuous shaking, and bacterial growth was monitored by recording the OD_{540} every 10 minutes for 12.5 hours.

Statistical Analysis of Bacterial Growth

Bacterial growth was monitored by measuring optical density at 578 nm (OD_{578}) over 750 minutes. The antimicrobial effect was quantified as growth-curve inhibition rather than endpoint MIC values. The Area Under the Curve (AUC) was utilized as the primary metric for statistical comparison to capture the integrated effect of the compounds on the total bacterial growth cycle. For this, the AUC was calculated for each replicate using the trapezoidal rule (MESS package in R). For statistical significance, a one-way Analysis of Variance (ANOVA) was performed followed by a Dunnett's post-hoc test to compare the AUC of each treatment concentration against the untreated control (0 µg/mL). All analyses were conducted in R (v. 4.4.2) within the RStudio integrated development environment (version 2024.09.1 Build 394, 'Cranberry Hibiscus' release). P-values were adjusted for multiple comparisons, and significance levels were defined as: $p < 0.05$ (*), $p < 0.01$ (**), and $p < 0.001$ (***).

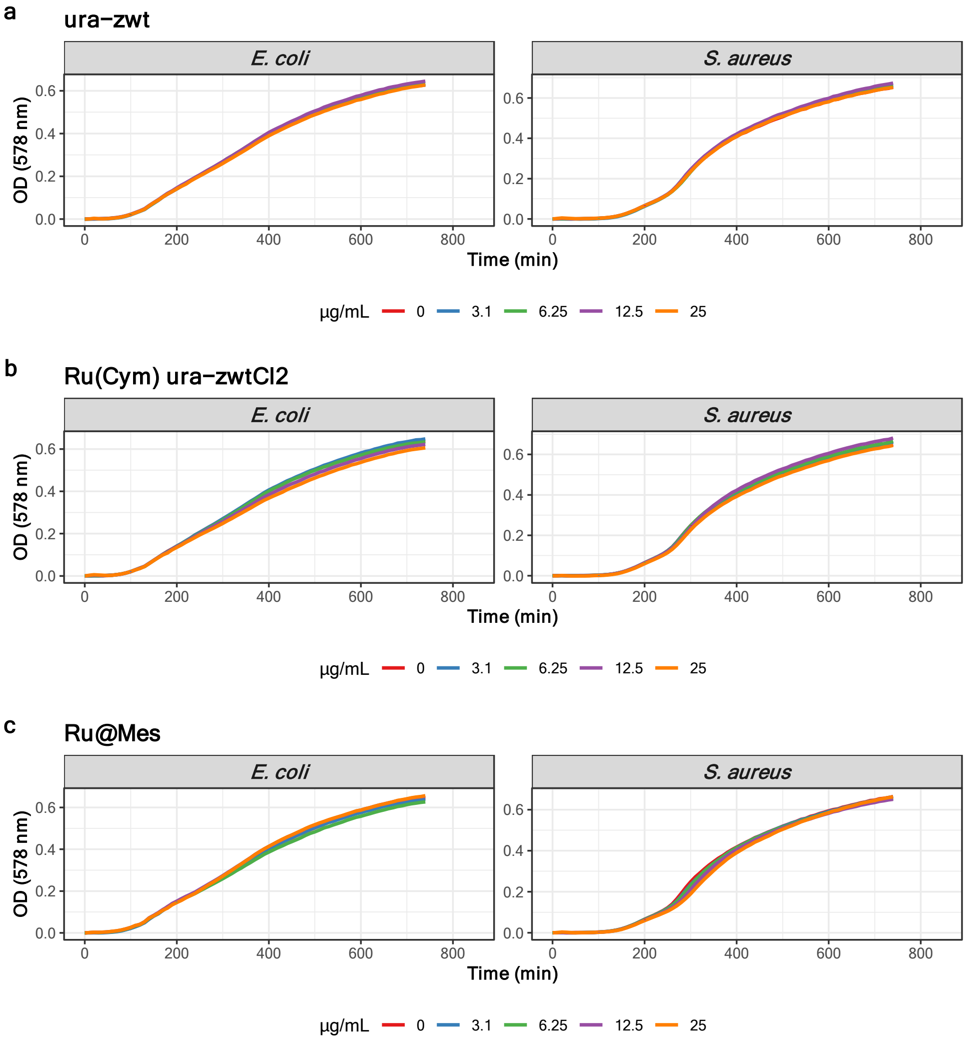

Figure S19. *Growth kinetics of E. coli and S. aureus treated with varying concentrations (0-25 µg/mL) of ura-zwt, Ru(Cym) ura-zwtCl, and Ru@Mes. No statistically significant differences between the treated groups and the untreated control, as determined by a one-way ANOVA followed by Dunnett's post-hoc test were found; n=2.*
